# *Hydrangea arborescens* ‘Annabelle’ Flower Formation and Flowering in the Current Year

**DOI:** 10.3390/plants12244103

**Published:** 2023-12-07

**Authors:** Xiaoxu Huang, Tong Lyu, Zheng Li, Yingmin Lyu

**Affiliations:** 1 Beijing Key Laboratory of Ornamental Plants Germplasm Innovation & Molecular Breeding, China National Engineering Research Center for Floriculture, College of Landscape Architecture, Beijing Forestry University, Beijing 100083, China; 2 Beijing Flower Engineering Technology Research Center, Plant Institute, China National Botanical Garden North Garden, Beijing 100093, China

**Keywords:** *Hydrangea arborescens*, transcriptome (RNA-seq), current-year flower development, phytohormone, gene co-expression network

## Abstract

The perennial woody plant *Hydrangea arborescens* ‘Annabelle’ is of great research value due to its unique mechanism of flower development that occurs in the current year, resulting in decorative flowers that can be enjoyed for a relatively long period of time. However, the mechanisms underlying the regulation of current-year flower development in *H. arborescens* ‘Annabelle’ are still not fully understood. In this study, we conducted an associated analysis to explore the core regulating network in *H*. *arborescens* ‘Annabelle’ by combining phenological observations, physiological assays, and transcriptome comparisons across seven flower developmental stages. Through this analysis, we constructed a gene co-expression network (GCN) based on the highest reciprocal rank (HRR), using 509 differentially expressed genes (DEGs) identified from seven flowering-related pathways, as well as the biosynthesis of eight flowering-related phytohormones and signal transduction in the transcriptomic analysis. According to the analysis of the GCN, we identified 14 key genes with the highest functional connectivity that played critical roles in specific development stages. We confirmed that 135 transcription factors (AP2/ERF, bHLH, CO-like, GRAS, MIKC, SBP, WRKY) were highly co-expressed with the 14 key genes, indicating their close associations with the development of current-year flowers. We further proposed a hypothetical model of a gene regulatory network for the development of the whole flower. This model suggested that the photoperiod, aging, and gibberellin pathways, along with the phytohormones abscisic acid (ABA), gibberellin (GA), brassinosteroid (BR), and jasmonic acid (JA), work synergistically to promote the floral transition. Additionally, auxin, GA, JA, ABA, and salicylic acid (SA) regulated the blooming process by involving the circadian clock. Cytokinin (CTK), ethylene (ETH), and SA were key regulators that affected flower senescence. Additionally, several floral integrators (*HaLFY*, *HaSOC1-2*, *HaAP1*, *HaFULL*, *HaAGL24*, *HaFLC*, etc.) were dominant contributors to the development of *H*. *arborescens* flowers. Overall, this research provides a comprehensive understanding of the dynamic mechanism underlying the entire process of current-year flower development, thereby offering valuable insights for further studies on the flower development of *H. arborescens* ‘Annabelle’.

## 1. Introduction

*Hydrangea arborescens* ‘Annabelle’ is a perennial woody plant native to eastern North America [1]. As one of the most cultivated members of the Hydrangeaceae family, *Hydrangea arborescens* currently contains nine cultivars, primarily cultivated as cut flowers and flowering pot plants. Among these cultivars, *H. arborescens* ‘Annabelle’ is valued for its ability to withstand cold climates, as well as its strong stems and stunning inflorescences [2]. The inflorescence forms of *H. arborescens* ‘Annabelle’ consist of round, densely branched corymbs with mainly showy decorative flowers (sterile flowers) bearing petaloid sepals. The diameter of large inflorescences can reach 25–30 cm. Apart from its ornamental value, *H. arborescens* ‘Annabelle’ demonstrates strong hardiness, exhibiting a wide range of resilience from hardiness zone 3 to zone 9 (USDA). It is capable of surviving temperatures as low as −40 °C [2,3]. *H. arborescens* ‘Annabelle’ can produce floral buds on both previous and current-year growth. Although low temperatures in winter and spring frosts cause damage or death to stems and flower buds formed during the previous season [4], the stable ability of current-year flower development in *H. arborescens* ‘Annabelle’ allows for an overlap of vegetative and reproductive growth. This characteristic enables the plant to regenerate new rhizome nodes in spring and continuously differentiate flower buds, resulting in the flower blooming punctually during summer. As a result, *H. arborescens* ‘Annabelle’ can be cultivated in open-field conditions without the need for protective measures [5,6]. Furthermore, *H. arborescens* ‘Annabelle’ is an excellent plant to function as cut flowers, because it has a unique trait that the decorative flowers remain open and retain their color long after the initiation of senescence. In conclusion, this plant exhibits an excellent mechanism of flower development that determines the trait of the current-year flower’s development. This mechanism enables a prolonged period of viewing, expands its application areas, and reduces the costs associated with maintenance [2,3].

Flower development is the most important event in the life cycle of ornamental plants that determines its ornamental and economic value. In the case of *H. arborescens* ‘Annabelle’, flower development involves four main steps. Firstly, floral induction occurs when the shoot apex meristem transitions from a vegetative state to a reproductive state. Secondly, flower morphogenesis takes place as the shoot apex differentiates into an inflorescence. Subsequently, blooming occurs as the decorative flowers’ sepals gradually unfold, reaching the full blooming stage. Finally, the inflorescence enters the senescent phase [7,8]. As the initial stage of flower development, floral induction determines the flowering time. Previous studies have extensively investigated the flower development mechanism in model plants like *Arabidopsis thaliana* [9], which provides a foundation for studying this process in perennials. Floral induction is influenced by various signals, including endogenous developmental cues and external environmental signals such as day length, temperature, and prolonged cold exposure [7]. These signals collectively form a complex gene regulatory network composed of the photoperiod, ambient temperature, and vernalization pathways in response to environmental changes, and the gibberellin, aging, and autonomous pathways are more susceptible to internal conditions [10,11,12,13]. It is important to note that these pathways do not independently regulate flower development, but rather, they interact with multiple genes and converge on common downstream target genes. These downstream target genes are often referred to as key floral integrator genes, including *FLOWERING LOCUS T* (*FT*), *SUPPRESSOR OF OVEREXPRESSION OF CO1* (*SOC1*), *FLOWER LOCUS C* (*FLC*), and *LEAFY* (*LFY*) [13,14,15,16]. They have both overlapping and distinct functions to encourage floral meristem characteristic genes, such as *LFY*, *APETALAI* (*AP*I), and *AGAMOUS-LIKE* (*AGL*), thereby promoting the whole process of flower development [17,18].

Phytohormones are crucial endogenous signals that play a vital role in regulating flower development. The regulatory mechanism of various hormones, including gibberellins (GAs), auxin, cytokinin (CTK), ethylene (ETH), abscisic acid (ABA), jasmonic acid (JA), salicylic acid (SA), and brassinosteroid (BR), primarily involve biosynthesis, transport, and signal transduction [19,20,21]. These phytohormones interact with each other in a synergistic or antagonistic manner, and ultimately influence the lifespan of flowers [22,23]. Gibberellins (GAs) are plant hormones involved in the regulation of flower development in response to endogenous and environmental signals, playing positive and negative regulatory roles in woody and model annual plants, respectively [23]. In *Arabidopsis thaliana*, the spatial precision of floral initiation is largely determined by the interplay between two types of phytohormones: auxin and CTK [19]. Additionally, the GA and CTK signaling pathways are connected through the DELLA protein [20]. ABA has varying effects on flower bud differentiation in different plants. It acts as a positive regulator in *Chrysanthemum morifolium* and *Annona squamosa*, while it inhibits bulblet formation in *Lycoris radiata* [21,24,25]. The interplay between different phytohormones in flower senescence have been extensively reviewed. ETH, ABA, and SA are recognized as key hormones that play an essential role in flower senescence [22,26]. Both ABA and IAA can accelerate the biosynthesis of ETH, leading to senescence. However, the endogenous content of CTK is negatively associated with petal senescence [22]. CTK treatment has not only delayed flower senescence in carnations [27], but also reduced ethylene production. Additionally, recent research has highlighted the role of novel phytohormones. JA participates in the development of floral organs and inhibits *FT* expression [28]. An exogenous application of MeJA can delay flower senescence [15]. SA is known as a negative regulator of the flowering suppressor *FLC* gene, indicating that it is a valuable phytohormone promoting flower development [29]. Previous studies have found that BR affects the flowering pathway in various ways, including partially independent effects on *FT* and *SOC1* expression [30]. However, the specific regulatory mechanism still requires further exploration. While it is well established that these hormones regulate flower development, the molecular mechanism by which various plant hormones are involved in the current-year flower development of *H. arborescens* ‘Annabelle’ remains largely unknown.

Flowers play a crucial role in determining the ornamental and economic value of horticultural plants. Plants that bloom for a long duration is the goal of gardeners. However, the genetic mechanism underlying current-year flower development in *H. arborescens* ‘Annabelle’ remains unknown and requires further exploration. In this study, we conducted phenological observations to characterize the current-year flower development in *H. arborescens* ‘Annabelle’ and identified seven key stages in the developmental process. Next, we performed a comparative transcriptome analysis of these flower development stages and investigated two categories of candidate genes involved in flowering-related pathways, as well as flowering-related phytohormones. To gain a deeper understanding of the functional relationships between genes, we constructed a gene co-expression network (GCN) and identified gene function modules associated with specific current-year flower development stages. This network provides valuable insights into the underlying molecular mechanism of the whole current-year flower development system.

## 2. Results

### 2.1. Phenotypic Observation in the Current-year Flower Development of H. arborescens ‘Annabelle’

**Figure 1:**
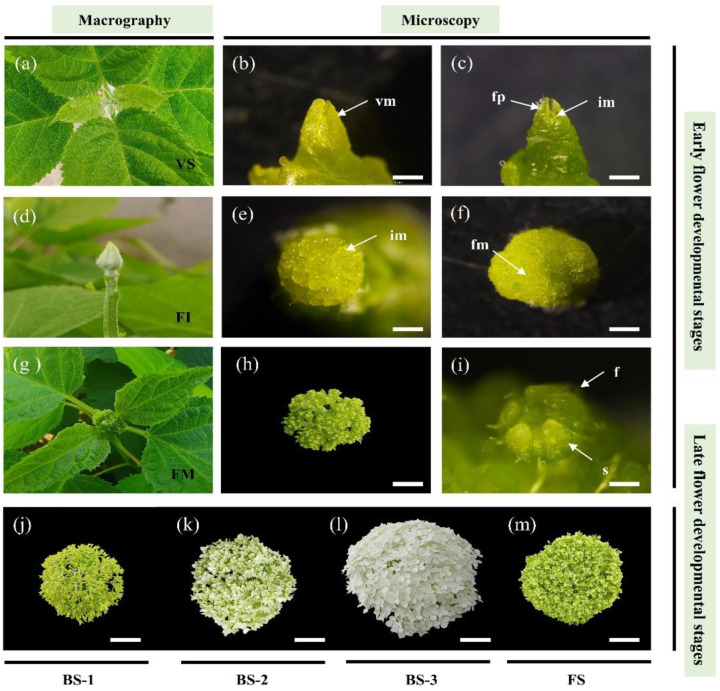
Flower development stages of current season’s shoots in *H. arborescens* ‘Annabelle’ observed through macrography and microscopy. (**a**) VS: vegetative bud, corresponding to Figure 1b (20 March). (**b**) Microscopic vegetative bud (20 March). (**c**) Inflorescence meristem dome formation (7 April). (**d**) FI: Flower bud expanded after differentiation, corresponding to Figure 1e (15 April). (**e**) Formation of numerous inflorescence meristem domes (15 April). (**f**) Differentiation of floral primordia (21 April). (**g**) FM: secondary branching elongation of spherical inflorescence via macrography, corresponding to Figure 1h,i (7 May). (**h**) Flower morphogenesis via microscopy (7 May). (**i**) Floret organ differentiation (7 May). (**j**) BS-1: early blooming stage (20 May). (**k**) BS-2: transitional stage of blooming (26 May). (**l**) BS-3: full blooming stage (1 June). (**m**) FS: flower senescence (10 July). vm = vegetative meristem; im = inflorescence meristem; fm = floral primordia; fp = foliar primordia; f = floret; s = sepal. Scale bars: (**b**,**c**,**i**): 0.5 mm; (**e**,**f**): 1 mm; (**h**): 1 cm; (**j**,**k**,**l**,**m**): 5 cm.

To characterize flower development, observations were initiated in March 2022 (Beijing, China). Plants were sampled from early March to early July, encompassing a period during which ten morphologically distinct stages were observed (Figure 1). During winter, the above-ground parts of the plants died due to the cold climate. However, the rhizome of *Hydrangea arborescens* ‘Annabelle’ could regenerate vigorous buds and maintained vegetative growth during March (Figure 1a,b). When the shoots had an average of five nodes per plant, there were no noticeable morphological differences in the newly formed apex buds, while microscopic observation revealed the presence of inflorescence meristem domes, indicating the completion of the transition from vegetative to reproductive growth (Figure 1c). Subsequently, significant bud swelling was observed (Figure 1d), and numerous inflorescence meristem domes were differentiated (Figure 1e), resulting in a distinctive global shape [31]. The inflorescence meristem then differentiated into the floral meristem (Figure 1f). The morphogenesis of secondary inflorescence branches (Figure 1g) and the differentiation of floral organ primordia occurred in early May (Figure 1i). The growth of the inflorescence axes continued until the fifth axes were fully formed. At this stage, the inflorescence entered the early blooming stage, with 10 nodes of current basal shoots (Figure 1j). The transition from the early blooming stage to the full blooming stage lasted for 5 days, during which the decorative floret sepals began to change color (Figure 1k). The full blooming stage lasted approximately 20 days, from early June to late June, with the inflorescence reaching a size of 25–28 cm and displaying a showy color (Figure 1l). Subsequently, flower senescence, the final stage of flower development, occurred. The decorative floret sepals completely changed color to deep green and remained colored for about one month, with ornamental value (Figure 1m). The plants started flowering in June and continued to bloom until July. The persistent decorative florets further prolonged the viewing period of *H. arborescens* ‘Annabelle’.

### 2.2. Quality Analysis of Transcriptome Sequencing

To further investigate the transcriptional mechanism of current-year flower development in *H. arborescens* ‘Annabelle’, we assessed morphological changes at different stages of flower development and selected seven representative stages for transcriptome sequencing (Table 1): vegetative stage (VS), floral initiation (FI), floral morphogenesis (FM), early blooming stage (BS-1), middle blooming stage (BS-2), full blooming stage (BS-3), flower senescence (FS). Total RNA was extracted from each stage to construct cDNA libraries, and Illumina RNA-seq was used for a comparative transcriptional sequencing analysis. VS-FM represented the early flower developmental phase, and BS-1-FS represented the late flower developmental phase.

The RNA sequencing of each sample yielded raw data of over 6 GB with a Q30 score higher than 93%. After filtering, the high-quality clean data obtained were no less than 6 GB. The GC percentage was approximately 45%, and the Q30 base distribution ranged from 93.85% to 94.53% (Table S1). These results indicated that the transcriptome sequencing was of high quality and met the criteria for subsequent assembly and data analysis. The assembly of the reads generated a total of 72,614 unigenes, with an average length of 1043 bp and an N50 length of 1828 bp (Table S2). All the unigenes were annotated using BLAST with an E-value < 0.00001. In total, 35,109 (48.35%) unigenes were annotated in the Nr database, while 34,367 (47.33%), 20,697 (28.50%), and 25,616 (35.28%) were annotated using the KEGG, KOG, and Swiss-Prot databases, respectively (Table S3).

### 2.3. Comparative Transcriptomic Analysis of Seven Flower Developmental Stages

**Figure 2:**
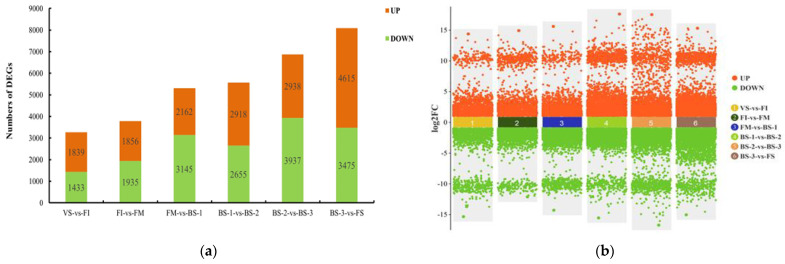
Distribution of DEGs in the transcriptional comparisons. (**a**) Histogram illustrating the numbers of up-regulated (orange bars) or down-regulated (green bars) DEGs in each pairwise comparison. (**b**) The X-axis represents each comparison, while the Y-axis indicates the log2 value of fold changes; orange spots represent up-regulated DEGs and green spots indicate down-regulated DEGs.

An analysis of differentially expressed genes (DEGs) was performed between each pair of adjacent stages (VS/FI, FI/FM, FM/BS-1, BS-1/BS-2, BS-2/BS-3, and BS-3/FS). DEGs with a *p*-value (FDR) ≤ 0.05 and a |log2Fold Change| ≥ 1 were considered significantly DEGs. As shown in Figure 2, the number of DEGs gradually increased during flower development. In the comparison of VS vs. FI, 1839 DEGs were up-regulated and 1433 DEGs were down-regulated. In the FI vs. FM comparison, 1856 up-regulated and 1935 down-regulated DEGs were detected. The comparison of FM vs. BS-1 revealed 2162 up-regulated and 3145 down-regulated DEGs. The number of DEGs continued to increase in later comparisons of flower development (Figure 2b). In the BS-1 vs. BS-2 comparison, 2918 DEGs were up-regulated and 2655 DEGs were down-regulated. At the full blooming stage (BS-2 vs. BS-3), 2938 DEGs were up-regulated and 3937 DEGs were down-regulated. In the period from BS-3 to FS, which means entering flower senescence, the total number of DEGs reached its peak, with a significantly higher number of up-regulated DEGs compared to down-regulated DEGs.

We further performed Gene Ontology (GO) and KEGG functional annotation to investigate the specific functions of all DEGs obtained through transcriptional comparisons. Utilizing the GO classification system, the DEGs were annotated with 52 functional terms, which were classified into three ontologies: biological process (BP), cellular component (CC), and molecular function (MF). In terms of biological processes, the major DEGs were related to cellular processes, metabolic processes, and biological regulation. Within the cellular component category, the top three gene groups were associated with cellular anatomical entities, protein-containing complexes, and virion components. In the molecular function category, most of the GO terms were grouped into binding, catalytic activity, and transporter activity (Figure S1). Additionally, the KEGG dataset was used to classify DEGs based on the pathways they were associated with or the functions they performed. A total of 8439 DEGs were assigned to 139 significantly enriched pathways in five branches: metabolism, genetic information processing, environment information processing, cellular processes, and organismal systems (Figure S2).

### 2.4. Identification of Candidate DEGs Involved in Current-Year Flower Development

To investigate flowering-related pathways, as well as the flowering-related endogenous hormones of current-year flower development in *H. arborescens* ‘Annabelle’, we identified candidate differentially expressed genes (DEGs) from significant GO terms, significant KEGG enrichment pathways, and other genes that showed significant differences or high expression levels in six comparisons to establish the core gene co-expression network (GCN). The enrichment analysis revealed the top 20 significant terms for GO enrichment (Figure S3) and the top 20 significant pathways for KEGG enrichment (Table S4) that were the most represented in each comparable group.

We observed that “DNA-binding transcription factor activity”, “transcription regulator activity”, and “regulation of nucleic acid-templated transcription” were common across all stages of flower development (Figure S3), suggesting that transcription factors (TFs) played a crucial role in plant growth and development [31]. Furthermore, the “Plant hormone signaling pathways” were found to be important for current-year flower development, as it significantly enriched in most of the KEGG analysis groups (Table S4). During the transition from vegetative growth to reproductive growth (VS to FI), we identified unique GO terms such as “shoot system morphogenesis” and “flower development” (Figure S3a). The KEGG analysis revealed that pathways related to the biosynthesis of BR, GA, IAA, and ABA were highly significant, such as the “brassinosteroid biosynthesis”, “diterpenoid biosynthesis”, “tryptophan metabolism”, “Zeatin biosynthesis”, and “Carotenoid biosynthesis” pathways, indicating their importance for the transition (Table S4). In the comparison of FI vs. FM, we observed several GO terms related to “floral whorl development”, “floral organ development”, and “reproductive shoot system development” (Figure S3b). These very highly significant terms suggested that this phase played a critical role in the differentiation of floral buds. The KEGG analysis further confirmed that many DEGs associated with the biosynthesis of ETH, SA, GA, ABA, and auxin were significantly enriched in this phase, such as “Cysteine and methionine metabolism”, “Phenylpropanoid biosynthesis”, “Plant hormone signal transduction”, and “Carotenoid biosynthesis”.

In the late flower developmental stage (FM vs. BS-3), DEGs associated with transcription activity-related processes were found to be the most significant terms (Figure S3c). Additionally, we conducted a KEGG pathway analysis and identified several hormone biosynthesis genes related to auxin, SA, GA, ABA, and CTK. During the blooming period, which included two comparison groups (BS-1/BS-2, BS-2/BS-3), the top 20 significant terms from the GO enrichment were related to cell expansion and energy metabolism, indicating the importance of cell walls in the blooming process and the gradual maturation of flowers [7,32]. “Phenylpropanoid biosynthesis” was a very highly significant KEGG pathway enriched in BS-1 vs. BS-2, and it was important for SA biosynthesis. In addition, the KEGG analysis revealed that the metabolic pathway of ETH and the biosynthetic pathway of ABA deserve attention. For flower senescence (BS-3 vs. FS), the most highly significantly enriched genes in senescent flowers were associated with their oxidoreductase activity. Surprisingly, senescence-associated endogenous hormones’ DEGs were selected, indicating that the interaction between ETH, ABA, IAA, and CTK probably mediates flower senescence.

Based on the aforementioned analysis results, a total of 509 candidate DEGs were identified to construct a GCN during flower development (Table S5). Some of the DEGs belonged to six important flowering-related pathways, including the photoperiod (64 genes), gibberellin (31 genes), ambient temperature (four genes), vernalization (two genes), age-related (19 genes), and autonomous (14 genes) pathways, and they also included floral integrators (three genes) and floral repressors (nine genes). Furthermore, the above analysis revealed that phenotypic changes occur during current-year flower development, involving the regulation of eight endogenous hormones and the up- or down-regulation of numerous genes in various phytohormone pathways. Notably, we have identified 346 DEGs in total related to the biosynthesis and signal transduction of hormones that are mainly distributed in the pathways of gibberellic acid (GA), auxin, cytokinin (CTK), brassinosteroid (BR), ethylene (ETH), abscisic acid (ABA), jasmonic acid (JA), and salicylic acid (SA).

### 2.5. Construction of Gene Co-Expression Network (GCN) and Detection of Functional Modules

**Figure 3:**
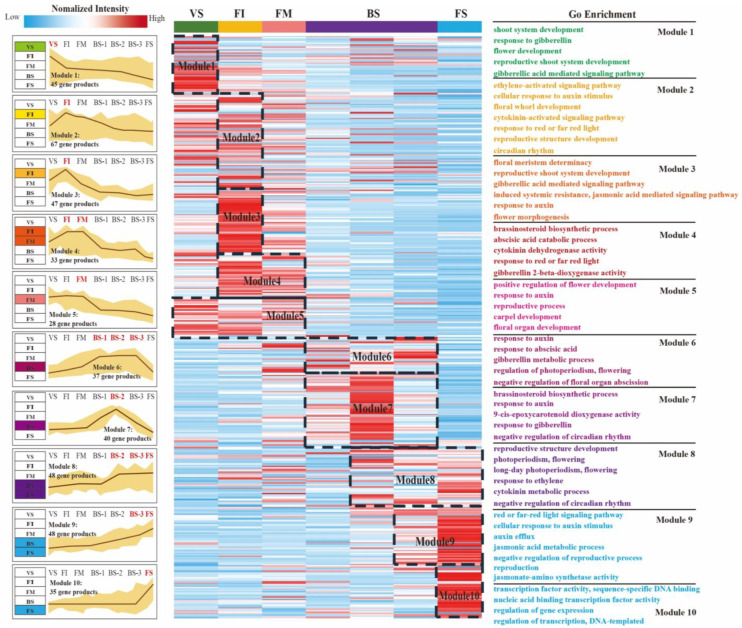
Ten modules in GCN reveal biological functional characteristics based on the process of flower development. Middle part: A heatmap of candidate DEGs’ expression trend during the flower development process. Each column within green, yellow, pink, purple and blue bars correspond to the developmental stages of flowers in *H. arborescens* ‘Annabelle’, respectively. The red to blue boxes indicate high to low expression levels. Left panel: Visualizations of the gene expression profile were helpful to reveal the co-expression patterns in ten modules. Significantly enriched GO categories (*p*-value ≤ 0.05) in each module are listed on the right side. The expression of candidate genes at all stages of flower development is normalized and displayed.

To understand the possible regulatory relationships of flower development-related genes, we constructed a gene co-expression network (GCN) using 509 identified DEGs involved in flowering-related pathways and phytohormone pathways. The construction of the GCN was performed using the highest reciprocal rank (HRR)-based method, and biologically related clusters were detected using the MCL algorithm [33]. Detailed information on the network construction can be found in Table S6. After removing meaningless clusters, a gene co-expression network with 14 biologically meaningful clusters was generated, which ranged in size from 6 to 67 genes per cluster (Figure S4). Table 2 provides the simple parameters of the constructed gene co-expression network. Based on gene expression profiling, we analyzed the gene expression characteristics of different clusters, and then formed ten functional modules with significantly different expression profiles (Figure 3). The expression profiles and enrichment results of each module at different flower development stages are presented in Figure 3. Finally, we characterized the functional properties of each module through differential expression displays, Gene Ontology (GO) enrichment (Table S7), and searches within the literature. These modules exhibited regular regulation patterns, suggesting that they hold biological co-expression relationships during the specific phase of flower development.

Module 1 included cluster 4. The expression levels of most genes were highest in VS, while there was a significant change from VS to FI. The most abundant GO term indicated that transcription factors (TFs) played a critical role in controlling vegetative growth (Table S7).

Module 2 included cluster 1 and cluster 11. The expression trend in this module showed an up-regulation from VS to FI, reaching a peak at FI. This trend indicated that this module affected the development of floral initiation (FI).

Module 3 included cluster 8 and cluster 12. The expression profile of these DEGs peaked during the FI stage. Upon entering flower morphogenesis (FI-FM), a significant down-regulation was observed. This result indicated that the genes related to this module might only play a key role in floral initiation (FI).

Module 4 included cluster 7. The expression level of Module 4 remained consistently high from the FI stage to the FM stage, indicating that this module was closely associated with floral organ development and floral morphogenesis.

Module 5 included cluster 9. Most DEGs maintained high expression levels throughout the early flower development stages (VS-FM), but significantly decreased during the blooming phase. This trend indicated that this module affected the entire early flower developmental phase.

Module 6 included cluster 5. The expression profile of these DEGs exhibited a significant change from FM to BS-1, with high levels maintained throughout the entire blooming period (BS-1-BS-3). It could be speculated that this module played a crucial role in promoting blooming.

Module 7 included clusters 10, 13, and 14. The genes exhibited a consistent increase from the FM stage to BS-2, but notably declined to a low level at the FS stage. The results suggest that genes within this module might influence the full blooming process (BS-2).

Module 8 included cluster 3. Most genes showed significant up-regulation from BS-1 to BS-2, and then maintained a stable expression trend at a higher level. Therefore, we could infer that these genes within Module 8 played a regulatory role in the sepal expansion.

Module 9 included cluster 2. Their expressions were continuously up-regulated throughout the entire later flower development of *H. arborescens* ‘Annabelle’, peaking at the FS stage. Thus, it was speculated that this module might influence the duration of blooming.

Module 10 included cluster 6. The expressions levels of these genes were firstly low during flower development and then significantly increased during flower senescence. This result suggests that the genes in Module 10 likely had critical functions in the process of flower senescence.

### 2.6. Analysis of Modules Highly Correlated with the Early Flower Developmental Phase

As discussed above, most identified DEGs exhibited a wave-like expression trend with alterations or peaks occurring at different time points. These DEGs might be crucial to the development of the *H. arborescens* ‘Annabelle’ flower at a specific stage or during a continuous process. Therefore, we focused on modules that showed high correlations with developmental stages and divided them into two groups: the early flower developmental phase (before blooming, VS-FM) and the late flower developmental phase (blooming and flower senescence, BS-1-FS). The analysis revealed a total of 220 co-expressed genes clustered into Modules 1, 2, 3, 4, and 5 during the early flower developmental phase (VS/FI and FI/FM) (Table S8). This period encompassed important developmental changes, such as floral induction, flower bud differentiation, and the establishment of floral morphogenesis, which were critical for determining the flowering time [32].

**Figure 4:**
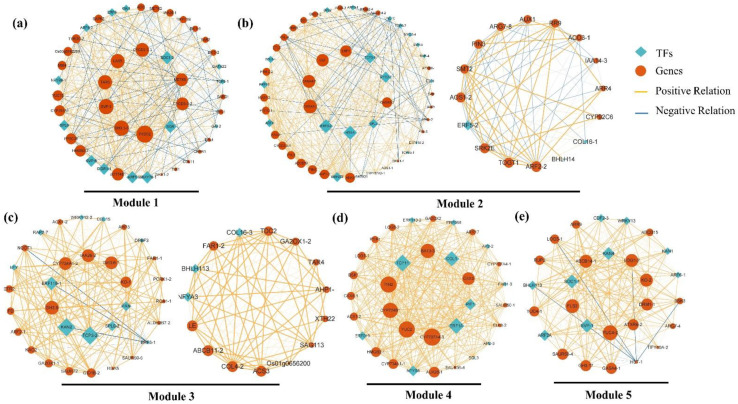
Predicted modules involved in the early flower developmental stages (Modules 1, 2, 3, 4, 5). Co-expression networks were visualized using *Cytoscape.* (**a**) Module 1; (**b**) Module 2; (**c**) Module 3; (**d**) Module 4; (**e**) Module 5. The size of nodes indicates the number of interacting DEGs; green nodes represent transcription factors (TFs) and red nodes represent genes. The width of the connecting lines indicates the strength of the correlation; solid lines indicate positive correlations and dashed lines indicate negative correlations.

Module 1 (Figure 4a) was found to be closely related to vegetative growth. We identified several GA signaling components (Table S8), including three transcripts encoding homologs of DELLA proteins (*GAI1*, *GAI-2*, *SCR*) and a GA-responsive protein (*CIGR1*). The role of auxin in controlling vegetative growth was also important, as evidenced by the presence of several hub genes involved in auxin biosynthesis and transport, such as *TYDC*, *GH3.1-3*, *TAR3*, and *LAX5*. Genes involved in ABA biosynthesis (*CYP707A1*, *AOG*) were detected, along with important floral integrators *SVP-3* and *SOC1-2* grouped in this module, which showed high expressions from VS to FI.

In Module 2, we identified the function of flowering-related pathways, such as the important gene in the autonomous pathway, *CLF*; the aging pathway gene, *SPL2/6-1/7*; and the photoperiod pathway, *COL16-1,* suggesting their potential roles in promoting floral initiation. Module 2 revealed that two ethylene response factors (*ERF3-2*, *ERF003*) were induced as hub genes, with two cytokinin receptors (*AHP1-2*, *AHK4*) and three components of the GA signal transduction (*GASAs*) also identified in this module. In Module 3 (Figure 4c), important mediators of the photoperiod pathway and aging pathway, such as *TCP3-2*, *COL4-2*, *FD*, and *SPL6-2,* were detected as intramodular hub genes. *LFY*, a key integrator of flowering signals, was found to be positively feedback-regulated by *SPL6-2* and *GH3.9* (Figure 4c). Additionally, hub genes *KAN2*, *GH3.9/3.6-1*, and *IAA26-2* play important roles in the auxin signaling pathway. The analysis of Module 3 also highlighted the involvement of GA biosynthesis genes (*KO-1*, *KAO2*, *GA2OX1-1*) and GA signal transduction genes (*GID1B-2*, *RGA1-1*).

The GO enrichment analysis of Module 4 indicated that this stage might be influenced by the photoperiod pathway, as well as endogenous hormones, including BR, ABA, and CTK. Within this module, *COL1* and *FTIP1,* known as positive regulators of the photoperiod pathway, were grouped together. The key gene *CYP707A4-3* was involved in ABA biosynthesis. Other intramodular hub genes such as *CYP734A1-1* and *CYP724B1* were both associated with BR biosynthesis. Additionally, important CTK biosynthesis-related genes (*CKX3*, *CKX5-1*) were also grouped in Module 4.

The top significant terms in Module 5 revealed that auxin played a crucial regulatory role in early flower development. This module consisted of numerous auxin-related genes, including the auxin biosynthesis limiting enzyme, *YUC4s*; the auxin efflux transporter, *ABCB14*; and the negative regulator factors, *KANs*.

The above findings imply that the photoperiod pathway, aging pathway, and gibberellin pathway were important in regulating the transition from vegetative growth to floral initiation. As described above, the early flower development phase was a sophisticated process in which phytohormones synergistically regulated most stages of flower development. During the floral transition, genes associated with auxin, ABA, and GA were particularly highlighted. CTK and BR also participated in the regulatory network during floral morphogenesis.

### 2.7. Analysis of Modules Highly Correlated with the Late Flower Developmental Phase

**Figure 5:**
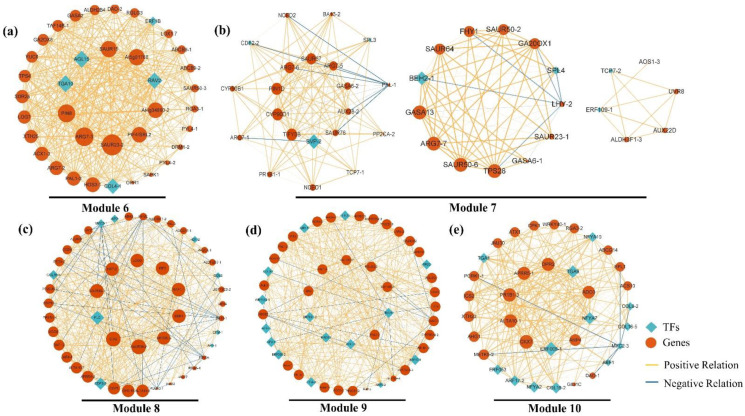
Predicted modules involved in the late flower developmental stages (Modules 6, 7, 8, 9, 10). Co-expression networks were visualized using *Cytoscape*. (**a**) Module 6; (**b**) Module 7; (**c**) Module 8; (**d**) Module 9; (**e**) Module 10. The size of nodes indicates the number of interacting DEGs. Green nodes represent transcription factors (TFs); red nodes represent genes. The width of the connecting lines indicate the strength of the correlation; solid lines indicate positive correlations and dashed lines indicate negative correlations.

The GCN analysis revealed 235 co-expressed genes grouped into Modules 6, 7, 8, 9, and 10 (Figure 5), which were correlated with the late flower developmental stages (BS-1, BS-2, BS-3, and FS). Investigating these gene expression modules and their potential molecular mechanisms was crucial in understanding the duration of blooming and flower senescence.

Several hub genes in Module 6 (Figure 5a) were annotated as auxin-transport genes, including *SAUR23-2*, *SAUR15*, *ARG7-2*, and *PIN8.* Notably, important genes involved in SA biosynthesis *(PAL1-3, TGA10)* were also identified in this module.

Most members of Module 7 (Figure 5b) showed significant up-regulation during FM to BS-1. The enrichment analysis indicated a strong correlation between this module and BR biosynthesis genes (*CYP70D1*, *CYP90B1*), and ABA biosynthesis genes (*NCED1*, *NCED2*, *PP2CA-2*) were also strongly correlated with this module. *SPL4,* a critical transcription factor in the aging pathway to promote flower development, was found to be up-regulated from FM to BS-2.

The most notable genes in Module 8 (Figure 5c) were primarily associated with SA, ETH, and JA. Phenylalanine ammonia-lyase genes (*PAL1-1*, *PAL1-2*, *PAL3-1*, *PAL3-2*) were involved in SA biosynthesis, with *PAL1-1* identified as the key gene. Additionally, one 1-amino cyclopropane carboxylic acid oxidase gene (*ACO3-2*) and two ethylene response factors (*ERF061-2*, *EIN3*) were identified. Notable genes related to JA biosynthesis genes (*JAR4-2*, *Acaa1b*, *Acox1*) were also found.

In Module 9 (Figure 5d), we investigated the involvement of auxin, ABA, and light signal-related genes in regulating the duration of blooming. The analysis revealed the presence of several auxin signal genes in this module, including *SAUR36-1*, *ABCB11-1*, *IAA17*, and *IAA3*. *SAUR36-1* was identified as the key gene in this module. Additionally, we identified seven ABA biosynthesis and signaling genes (*PP2CA-1*, *PP2C06-2*, *SDR1*, *RAF1.1*, *RAF1.2*, *CCD4*, *ABA2*) in this module. Some genes (*APRR5-2*, *COP10*) were involved in the circadian clock, which were presented in the most enriched term (Table S7). *FLC*, a flower repressor, integrates signals from the gibberellin pathway, photoperiod pathway, and aging pathway.

In Module 10 (Figure 5e), several genes related to ethylene biosynthesis (*ACS10*, *METK5-2*) and ethylene signaling transduction (*ERF008-1*, *ERF053*) were identified. Notably, *ERF008-1* (ethylene-responsive transcription factors) and *CKX7* (cytokinin dehydrogenase gene) were identified as hub genes. Furthermore, the core circadian clock gene (*APRR5-1*) and clock output gene (*ADO3*) expression were also considered as hub genes in Module 10.

Based on our major findings in the late flower developmental stages, we proposed that the circadian clock might be a central factor in regulating blooming, closure, and even senescence. Phytohormones, especially ABA, SA, and BR, were found to be important in regulating blooming initiation. Meanwhile, it also noteworthy that the co-regulation between JA, SA, and ETH might act as critical roles in the process of blooming. Surprisingly, in addition to ETH, we found that phytohormones such as ABA, CTK, auxin, and SA influenced the process of flower senescence synergistically or antagonistically.

### 2.8. Real-time Quantitative PCR (qRT-PCR) Validation of RNA-seq Data of Flower Development-Related Genes

**Figure 6:**
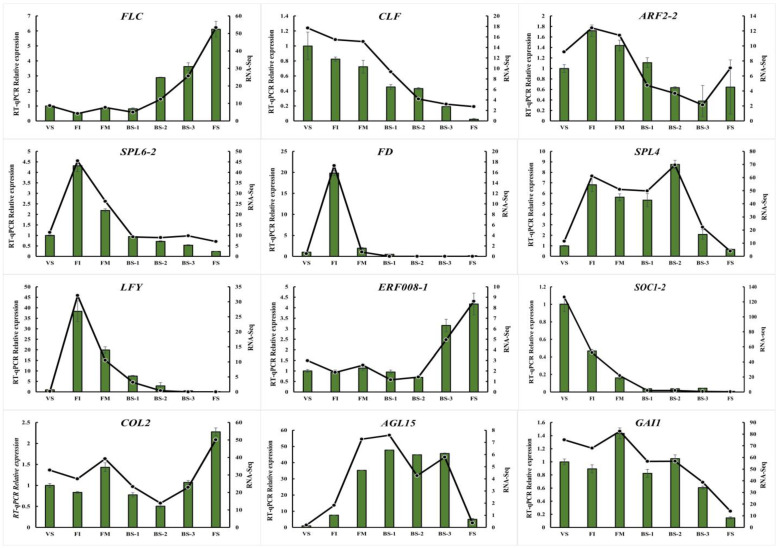
Validation of the expression of flower development-related genes via qRT-PCR analysis. Bar charts indicate values of qRT-PCR. Line plots indicate values of fragments per kilobase per million (FPKM). Error bars indicate the standard deviation of three biological replicates (n = 3).

To validate the accuracy of the transcriptome sequencing results, we randomly selected a total of 12 flower development-related unigenes (*FLC*, *CLF*, *ARF2-2*, *SPL4*, *SPL6-*2, *FD*, *LFY*, *ERF008-1*, *SOC1-2*, *COL2*, *AGL15*, *GAI1*) from the DEGs and detected them using qRT-PCR. We observed that the same change trends in the expression of these unigenes were shown between the qRT-PCR and fragments per kilobase per million (FPKM) values (Figure 6), and the Pearson’ s correlation coefficients of both were above 0.90, which indicates that the expression trends of most unigenes corresponded well between the two methods.

### 2.9. Changes in Phytohormone Content during Current-Year Flower Development

**Figure 7:**
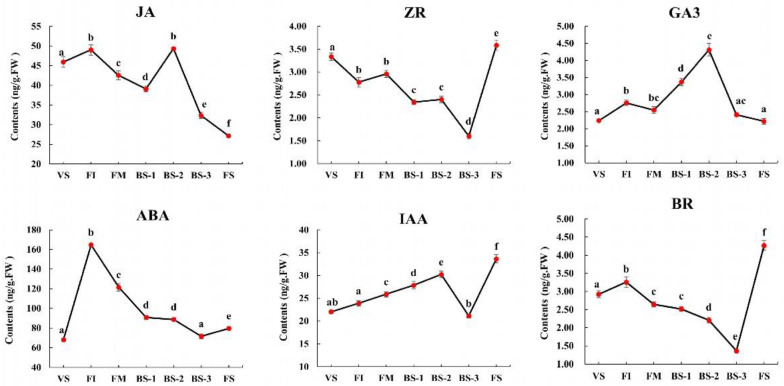
Changes in Indole-3-acetic acid (IAA), gibberellic acid 3 (GA_3_), abscisic acid (ABA), zeatin riboside (ZR), brassinosteroid (BR), and jasmonic acid (JA) contents during flower development. (a) IAA. (b) GA_3_. (c) ABA. (d) ZR. (e) BR. (f) JA. Data are shown as the mean ± standard error; different letters represent significant differences between the means (*p* < 0.05).

To investigate the role of phytohormones in *H. arborescens* ‘Annabelle’ flower development, we analyzed the contents of IAA (the most common auxin), ABA, GA_3_, ZR (a type of cytokinin), BR, SA, and JA at seven different developmental stages (Figure 7). Among these phytohormones, the contents of GA_3_, ABA, BR, and JA showed a significant increased during the FI stage, while the ZR content significantly decreased at this stage. The content of IAA continued to rise from VS to the BS-2 stage, while a sharp drop occurred to the lowest level at BS-3, and then reached a peak at FS. After reaching the highest level in BS-2, the content of GA_3_ significantly decreased during the full blooming phase (BS-3) and senescent phase (FS). Notably, ABA reached its highest content at FI, but decreased rapidly once entering the FM stage. After FI, we observed a similar trend between ZR and BR, with a slight decrease until BS-3, which then showed a significantly upward trend from BS-3 to FS. The levels of JA content fluctuated complicitly throughout the entire flower developmental phase, with the first peak occurring in FI, and the second peak occurring in BS-2, while a significant decline occurred from BS-2 to FS.

## 3. Discussion

### 3.1. Key Genes and Important Transcription Factors (TFs) Involved in Current-Year Flower Development

**Figure 8:**
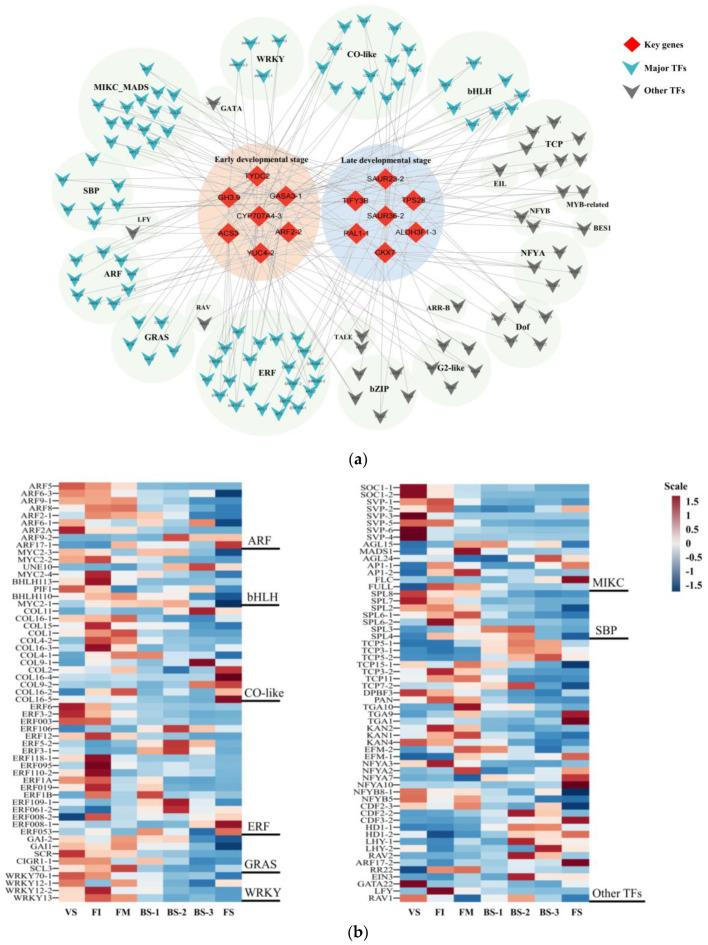
Distribution of the differentially expressed transcription factors (TFs) and co-expression networks between key genes and transcription factors. (**a**) Co-expression networks between differentially expressed key genes and differentially expressed TFs involved in the flowering-related pathway, phytohormone biosynthesis, and signal transduction pathway. (**b**) The expression profiles of the above TF families in different flower development stages.

Through the analysis of the GCN, each module was characterized by a key gene, which represents an important biological factor in promoting the development of flowers in *H. arborescens* ‘Annabelle’. Transcription factors are critical for a variety of processes related to plant growth and development. The co-expression of various TFs was identified with key genes. A model describing the regulation is proposed in Figure 8 to identify critical TFs involved in flower development. We detected the expression of 14 key genes with the highest functional connectivity in each module, namely *HaTYDC2*, *HaGASA3-1*, *HaARF2-2*, *HaGH3.9*, *HaACS3*, *HaCYP707A4-3*, *HaYUC4-2*, *HaSAUR23-2*, *HaTIFY3B*, *HaTPS28*, *HaALDH3F1-3*, *HaPAL1-1*, *HaSAUR36-2*, and *HaCKX7*. These genes were found to be highly regulated by 135 transcriptions factors (Figure 8a), including major TF families such as AP2/ERF, bHLH, CO-like, GRAS, MIKC, SBP, and WRKY, as well as other small families of TFs like GATA, TCP, NF-Ys, and bZIP, indicating that these TF families were likely to be involved in the regulation of flower development.

The *MADS-box* family, specifically the *MIKC-type MADS-box* transcription factors, have been shown to play key regulatory roles in various stages of flower development, including the regulation of flowering time and the formation of flower organs [34,35,36,37]. In this study, 15 *MIKC* TFs were found to be differentially expressed (*SOC1*, *SVP*, *AP1*, *FLC*, *AGL15/24*) and were mainly distributed in Module 1, Module 6, Module 8, and Module 9. This indicates that there was a tightly controlled balance between the activity and functioning of floral timing and the specification of floral organ identity. *MIKC* TFs played a significant role in the transition from vegetative to reproductive development, leading to a robust phase switch and the development of flowers and reproductive organs under optimal environmental conditions. *HaSOC1-2* showed high expression during early floral development, which supported previous reports that *SOC1* acts as a flowering activator by integrating signals from the photoperiod, vernalization, and GA pathways [37,38]. Furthermore, interactions among *MIKC* TFs are important for integrating flowering-related signals. For instance, *AGL15-SVP*, *AGL15-AGL24*, and *AGL15-SOC1* dimers are capable of blinding to the *SOC1*-specific promoter region [38,39].

Two *AP1s* (*HaAP1-1*, *HaAP1-2*) that were highly expressed in FI were identified. Previous studies have shown that *AP1* directly represses the flowering time genes *SVP*, *AGL24*, and *SOC1* in emerging floral primordia. Furthermore, it represses the members of the *SPL* family, promotes the transcription of *LFY* as part of a positive feedback loop, and controls the expression of floral homeotic genes. It is worth noting that a total of seven *SPLs* belonging to the SBP family were identified. Previous studies have reported that *SPL* TFs a play a major role in the aging pathway and serve as a key hub for integrating various flowering regulation pathways in *Arabidopsis* [40,41]. Based on this research, we found five members of the SBP family, namely *HaSPL2*, *HaSPL6-1*, *HaSPL6-2*, *HaSPL7*, and *HaSPL8*, which were all grouped into Module 2 and Module 3, suggesting that the aging pathway might play a central role in regulating current-year floral induction. Furthermore, *SPLs* directly bind to *SOC1* and *LFY* promoters, activating their transcription and regulating the initiation of floral meristems [42,43].

In this study, we identified 13 homologs (*HaCOLs*) of the *CONSTANS-like* (*COL*) family transcription factors in the *H. arborescens* ‘Annabelle’ transcriptome (Figure 8b). *CO-like* TFs are known to be regulated by the circadian clock and responses to changes in light signaling. They present throughout both the vegetative and reproductive phases [44]. There were four *COLs* (*HaCOL15*, *HaCOL16-3*, *HaCOL4-2*, *HaCOL1*) highly expressed in the early flower developmental stages, while *HaCOL9-2* and *HaCOL16-5* were significantly highly expressed in FS. This finding indicated that the members of the *COL* family regulate flowering in different ways, including promotion and repression. For instance, *CsCOL1* may work upstream of *CsFT*, acting as flowering activators under long day conditions in *Cymbidium sinense* [45], whereas *OsCOL16* acts as a constitutive inhibitor of flowering in rice [46]. Previous studies have demonstrated that *bHLHs* can regulate various processes related to flower development [47]. In our analysis, most of the *bHLH* TFs detected were related to JA signaling. Notably, *MYC2* is a well-known transcription factor that interacts with *JAZ* proteins and activates the transcription of early JA-responsive genes [48]. We observed the presence of three *HaMYC2* TFs (*HaMYC2-2*, *HaMYC2-3*, *HaMYC2-4*) grouped in Module 1 and Module 2, and their expression levels were particularly high during the early flower developmental phase.

Additionally, ARFs are important auxin response factors, regulating auxin response gene expression [49]. From Figure 8, it could be observed that three, two, and three *ARFs* were positively correlated with *HaTYDC2*, *HaGASA3-1*, and *HaYUC4-2*, respectively. This finding was consistent with the contents of auxin hormones and the GCN analysis, suggesting that auxin played an important role in mediating the flowering transition in *H. arborescens* ‘Annabelle’. In our work, we found that *GRAS* TFs were involved in GA signaling, such as *HaGAI1*, *HaGAI2*, *HaSCR*, and *HaCIGR1-1*. DELLA protein *GAIs* were found to display high expression levels in VS and were significantly down-regulated in FI. As the main repressors of GA responses, DELLA proteins play the most important role as the center regulatory nodes in the GA signaling transduction pathway [50]. Furthermore, *AP2*, a major subfamily of *AP2/ERF* TFs, has been found to regulate flower development, floral transition, and shoot apical meristem (SAM) maintenance in *Arabidopsis* through miR172-mediated regulation [51]. Two *AP2s* (*HaAP2-2*, *HaAP2-3*) were distributed in Module 4 and showed a high correlation with *HaCYP707A4-3*. As part of the *AP2/ERF* superfamily, *ERFs* play a key role in ETH signaling transduction, controlling the longevity of flowers and leaves by regulating the process of senescence and abscission [52]. Eleven *ERFs* were enriched in Modules 2, 3, 4, while only two *ERFs* (*HaERF008-1*, *HaERF053*) were significantly highly expressed during the senescent phase. We speculated that the low response of ethylene during flower senescence might change the ethylene response network [22], resulting in the long viewing period of *H. arborescens* ‘Annabelle’.

### 3.2. GA-, Aging-, and Photoperiod-Related Flowering Pathways Contribute to the Current-Year Flower Development of H. arborescens ‘Annabelle’

**Figure 9:**
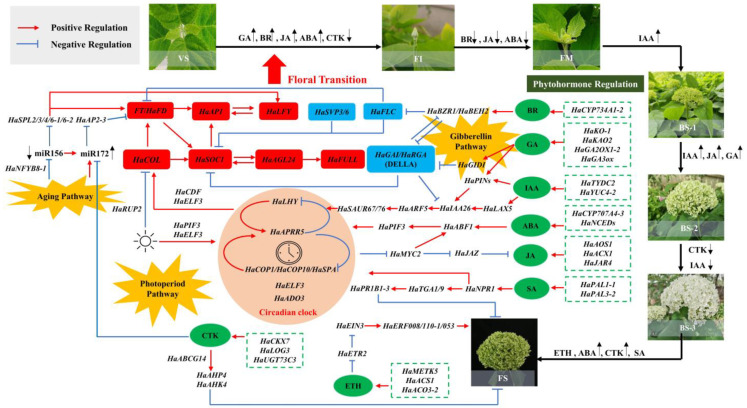
The proposed model of a gene regulatory network for the current-year flower development in *H. arborescens* ‘Annabelle’. The black arrow next to the various hormones represent the significant change of hormone content between two adjacent stages (*p* < 0.05).

According to the results we obtained in this study, the GA pathway, aging pathway, and photoperiod pathway were identified as important factors in the current-year flowering process of *H. arborescens* ‘Annabelle’. Previous reports have indicated that the aging pathway is partly mediated by GA and photosynthesis [8,53]. Genes involved in these pathways form a complex network of feedback loops and ultimately lead to the activation of floral integrators such as *HaLFY*, *HaSOC1-2*, *HaAP1*, *HaFUL*, *HaSVP,* and others. These integrators play a direct role in governing the flowering time. Based on the floral regulatory network in the model plant *Arabidopsis*, we developed a model depicting the molecular and genetic networks regulating the current-year flower development of *H. arborescens* ‘Annabelle’, as shown in Figure 9.

In the photoperiod pathway, genes such as *HaPHYE, HaPHOT1, HaUVR8,* and *HaFHY1* acted as light sensors and were highly expressed in the FI stage; they played critical roles in integrating environmental signals, and took them to the central circadian clock [53,54]. *HaELF3* and *HaPIF3* were responsible for transmitting signals to the downstream circadian clock [55]. It is well established that that the circadian clock is closely associated with the entire plant life cycle, particularly in regulating flower opening [56]. Our findings indicate that the core components of the central oscillator, such as *HaLHYs*, *HaAPRR5s*, *HaCOP1*, *HaCOP10*, and *HaSPA4*, exhibited regular expression patterns throughout the flowering process. This supports the fundamental roles of light and the circadian clock in flower opening. Previous studies have confirmed that the COP/SPA complex in the circadian clock can mediate flowering by regulating hormonal signaling [57]. Clock output genes, *HaCDF2/3,* were also depicted in a light-signaling network, suggesting the critical role of the circadian clock in flower development. By promoting the degradation of the *HaCDFs*, the *CO/CO-like (COL)* TF family [58], which are key transcription factors for photoperiodic flowering, can be active. They have dual roles that can be regulated by the circadian clock and directly promote the expression of downstream floral integrators, such as *FT*, *LFY*, and *SOC1*, thereby regulate flowering time. *HaCOL4-2*, *HaCOL15, HaCOL16-1*, and *HaCOL16-3* presented high expression levels during the early developmental phase and were significantly enriched in floral initiation-related modules (Modules 2/3). Other *HaCOLs* presented high expression during the full blooming phase; although they had little effect on the flowering time, their involvement in the period of circadian rhythms like the *AtCOL2* in *Arabidopsis* warrants further study [57,58,59]. As a major integrator of flowering-promoting pathways, *FT-like* proteins interact with the *bZIP* transcriptional factor *HaFD* (expressed in apical meristems) to form *FT/FD* complexes. *HaFD* and *HaFTIP1* (an essential regulator required for *FT* transport) were significantly up-regulated, indicating that the photoperiod played a critical role in floral induction. Following the formation of *FT/FD* (Figure 9), multiple changes were observed in the genes responsible for floral organ identification, including *HaAP1s* and *HaFULL*, thereby promoting the expression of *HaLFY*. Previous studies have shown that *FT/FD* regulates *AP1* indirectly by up-regulating the expression of *SOC1* during floral bud differentiation [60]. *SOC1* and *AGL24* have been found to interact and mutually enhance each other’s regulation, creating a positive feedback loop in young floral meristems [38].

The aging pathway plays a crucial role in regulating flowering in plants. During the juvenile phase, the aging pathway prevents flowering by maintaining a high level of microRNA156 (miR156). As plants transition into the adult phase, there is a gradual decline in miR156 abundance, accompanied by an increase in *SBP-LIKE* (*SPL*) transcription factors. This decrease in miR156 is also associated with the accumulation of miR172, which leads to the repression of *HaAP2s* transcription factors and the induction of *FT/FD*. The combined signals from miR172-*FT* and miR156 contribute to the activation of *SPL* genes, which are necessary for the expression of floral meristem identity genes [42,43]. *AtSPLs* can interact with *AtFT* and *AtSOC1* in the shoot apex meristem, leading to the expression of floral meristem identity genes *AtAP1*, *AtLFY*, and *AtFUL* [43]. Based on our findings, we observed that *HaSPL2/3/4/6-1/6-2* were significantly highly expressed in FI, with *HaSPL6-2* having been strongly correlated with *HaLFY*, *HaFD*, and *HaRGA5* (Figure 4), indicating that *HaSPL6-2* was a core factor in the floral network. We also noted that *HaAP2-3*, which is the target TF of miR172, exhibited a significant decrease in FI. Furthermore, previous studies have highlighted the role of *NF-YB* family genes as regulators in the photoperiod and GA pathways. *CmNFYB8* has been identified as a regulator that directly triggers miR156-SPL-regulated molecular processes, thereby influencing *chrysanthemum* flowering time [61]. We detected that *HaNFYB8-1/2* were highly correlated with *HaGAI1*, *HaSOC1-2,* and *HaSVP3/6*. GA activates its signaling pathway by binding to the receptor, *GID1*, and then promotes the degradation of GA-signaling repressors, the DELLA proteins [62]. Herein, we detected two types of DELLA proteins: *HaGAI1/2* and *HaRGA1/2/3/5*. The expression levels of these proteins gradually decreased throughout the developmental process. As gibberellin receptor genes, the expression levels of *HaGID1s* were significantly increased in the early stages of flower development, which enhanced the inhibition of DELLA proteins. DELLA proteins play a major role in mediating the interaction between GA and key regulators involved in other flowering genetic pathways or phytohormone signaling pathways. DELLA-*COP1* and DELLA-*CO/NF-YB* are both involved in GA-mediated *FT* regulation. Another study also shows that DELLAs directly interact with the strong flowering repressor *FLC* and promote its repressive function on downstream targets, including *FT* [63].

### 3.3. The Putative Roles of Phytohormone Crosswalk in the Current-Year Flower Development of H. arborescens ‘Annabelle’

According to above analysis in this study, we identified that phytohormones were important endogenous signals in regulating flower development, especially gibberellic acid (GA), auxin, cytokinin (CTK), brassinosteroid (BR), ethylene (ETH), abscisic acid (ABA), jasmonic acid (JA), and salicylic acid (SA). In our work, it was helpful for understanding the role of plant endogenous hormones during the specific developmental phase to reveal the changes in the content of various plant endogenous hormones and the expression of related genes throughout the development of *H. arborescens* ‘Annabelle’.

Associated with the determined phytohormone contents and the expression of genes related to biosynthesis and signaling of the above analysis, we found evidence supporting the important roles of GA_3_, auxin, ABA, BR, and JA in promoting flowering and regulating the floral transition and flower bud differentiation. In model plants like *Arabidopsis*, gibberellins have been shown to regulate floral induction through the GA pathway. The content of GA_3_ from FI to BS-2 was significantly higher than that in VS. Critical GA biosynthesis genes (such as *HaKO-1*, *HaKAO2*, *HaGA2OX1-2*, and *HaGA3ox*) showed higher expression levels during FI (Figure 4c) and were highly correlated with *HaLFY* and *HaFD*. These results suggest that GA activated flowering in *H. arborescens* ‘Annabelle’ by inhibiting the function of DELLA proteins [23] and promoting the expression of *HaLFY* and *HaFD*.

The role of auxin in plant flowering transition has been extensively studied; it has been reported to gradually concentrate in the SAM during the flowering transition, suggesting that auxin plays a key role in mediating this transition [64,65]. The endogenous auxin content and auxin biosynthesis genes (*HaTDC2*, *HaTYDC2, HaYUC4-2, HaALDHs)* were found to be maintained at higher levels during the early flower developmental phase than the late developmental phase. *HaTYDC2* and *HaYUC4-2*, which were key genes in Module 1 and Module 5, respectively, promote the synthesis of auxin (Figure 4 and Figure 8a). Through the identification of hub genes in the early flower developmental stages, we found the typical auxin signal transduction genes *HaLAX5*, *HaGH3.1-3/3.6/3.9,* and *HaIAA26-2* grouped in early-stage modules of flower development. This finding indicates that auxin was very important in flower morphogenesis and floral meristem formation, which is consistent with auxin signaling regulators being known for having a function in cell growth and expansion [64]. *ARF5* has been identified to be essential in determining floral primordia initiation and can activate floral meristem identity genes, such as *LFY* and *FUL* [65]. Meanwhile, GA can regulate *PIN-FORMED* (*PIN*) auxin transporters in *Arabidopsis thaliana* [66]. Auxin has been shown to regulate GA signaling and biosynthesis, such as *AUX/IAA* proteins negatively regulating the stability of DELLAs [67]. Notably, small auxin up-regulated RNAs (*HaSAUR67/76*) showed a significant enrichment from BS-1 to BS-3. These findings suggest that auxin might be involved in regulating cell elongation and the expansion of decorative flowers through the interaction between *SAUR* genes in the auxin response pathway and the circadian clock [68]. These biological processes are important in the flower opening process from BS-1 to BS-3.

Our results show that ABA was very important for the current-year floral initiation and flower senescence. Most ABA biosynthesis-related genes displayed the highest level in FI (*HaNCEDs*, *HaCYP707A4-1/3*, *HaAOG*, *HaTOG*, etc.) and then gradually decreased, which is consistent with the changes in ABA content. We further identified the function of ABA that promoted floral initiation and differentiation, because *HaCYP707A4-3* was the key gene in Module 4 and had positive correlations with several key photoperiod-related genes (*HaELF3-2*, *HaFTIP1*, *HaCOL16-3*, etc.). Additionally, our analysis identified *HaABF1*, an ABA-responsive transcription factor (*ABF*), which exhibited a high expression level from FM to FS. This result aligns with previous studies showing that *ABF* can modulate *SOC1* expression, thereby indirectly affecting *FT* transcription, and ABA is shown to promote flower senescence [7,69].

Compared to vegetative growth, the contents of BR, ETH, and JA were also significantly up-regulated in FI (Figure 7). The key gene responsible for BR biosynthesis, *HaCYP734A1-2*, was identified as a hub gene in Module 3 and displayed the same expression pattern as the change in BR content. In *Arabidopsis*, BR is critical for floral transition, inflorescence stem architecture formation, and other reproductive processes [70]. BR signaling may impact flowering in a GA-independent pathway, as the suppression of BR signaling reduced *FLC* expression. *BZR1* (BR signaling-related transcription factor) regulates the early stages of flower development by activating the expression of *SPL* and DELLA proteins. Moreover, our findings suggest that the JA content was significantly up-regulated in FI and BS-2, confirming its role not only in promoting floral transition, but also as a critical regulator of anther dehiscence in plants [71]. JA synthesis was positively regulated by several biosynthesis related genes, including *HaAOS1*, *HaAOC*, *HaACX1*, and *HaJAR4*. As the master transcription factor in JA signaling and one of the first TFs activated by JA, three transcripts of *HaMYC2s* were detected, and their levels continuously increased until BS-2. We hypothesized that the high transcriptional levels of *MYC2s* might be due to the release of the *JAZ1-MYC2* complex, which subsequently induced downstream genes related to female flower development, such as *LFY*, *FT*, and even the ABA signaling pathway [72]. Interestingly, *HaMYC2-3* exhibited a negative regulation of ETH biosynthesis genes, *HaMETK5-2*, in Module 10, suggesting that JA might delay flower senescence, but the role of JA in flower senescence is still unclear.

Phytohormones have been shown to play a pivotal role in triggering and modulating the progression of senescence, often through combinatorial interactions. As for phytohormones ETH, SA, and CTK, they were confirmed to be closely related to flower senescence in this study (Figure 5). We indirectly investigated ETH and SA’s involvements in current-year flower development by analyzing the expression of genes related to their biosynthesis and signaling. The analysis of the GCN revealed that ETH played a critical role in flower senescence, as evidenced by the high expression of several ETH biosynthesis genes (*HaMETK5*, *HaACS1, HaACO*, *HaACO3-2)* during the full blooming stage (BS-3). Moreover, *HaACO3-2* was a hub gene in Module 8, indicating the initiation of the senescence process only when the flowers enter the cell expansion phase [7]. However, the expression levels of most ETHYLENE RESONSE FACTORs (*ERFs*) were high in stage FI, whereas few *ERFs* (*HaERF110-1*, *HaERF008*, *HaERF053*) showed relatively high expression levels during flower senescence. Associated with a previous study on *Gypsophila paniculate* [22], we speculated that the reduction in downstream *ERFs* might lead to the ethylene insensitivity of *H. arborescens* ‘Annabelle’. This insensitivity affected the rate of flower senescence, allowing for a longer viewing period and the potential for dried flowers. Additionally, as important genes involved in SA biosynthesis [73], *HaPALs* were identified as the hub genes (*HaPAL1-1, HaPAL3-2*) in Module 8, indicating that SA was critical for the unfolding process of decorative flowers. In the process of SA signal transduction, *NPR1* acts as an SA receptor that regulates the transcription of core circadian clock genes in an SA-regulated manner. *HaTGA1/9* and *HaPR1B1-3* were found to be enriched in the senescent module (Module 10). These results are consistent with previous studies highlighting that SA might play a role in delaying flower senescence [74], but need further exploration.

Several studies have found that cytokinin regulates cell division and differentiation in floral meristems [75]. In our study, we proposed that CTK might inhibit floral transition. The content of CTK showed a significant down-regulation at the FI stage (Figure 7), which aligns with a previous study suggesting that CTK can activate the *AP2* protein by down-regulating the expression level of miR172, thereby inhibiting flowering [76]. Notably, CTK displayed a significant downward trend in BS-3, while showing an upward trend in FS. Associated with ETH biosynthesis genes being highly expressed in BS-3, we confirmed that flower senescence was initiated by an increase in ethylene production and a decrease in CTK levels [27]. Several studies have also demonstrated that CTKs prolong the life span of flowers in various plant species by delaying flower senescence [27]. In FS, we detected that numerous genes involved in CTK biosynthesis (*HaCKX7*, *HaLOG3*, *HaUGT73C3*) were highly expressed, resulting in an increase in CTK content. This finding is consistent with a previous study which reported that high levels of CTK in dividing cells act as the inhibitor of senescence [7]. As we all know, flower senescence is regulated by hormone crosstalk. For instance, ETH induces flower senescence by regulating CTK signaling and content [77]. However, the exact role of CTK in flower senescence and its interactions with other senescence-related phytohormones are complex and need further clarification in future research.

In conclusion, the interaction of endogenous GA, auxin, BR, ABA, and JA affected the floral transition process. GA promoted the expression of *FT/FD* by inhibiting the function of DELLA proteins and affected the transformation of flowers by regulating *HaLFY*. The flow of auxin in *H. arborescens* ‘Annabelle’ was also involved in the regulation of floral induction and floral morphologies. Auxin participated in the signal transduction process of GA by promoting the expression of *HaPINs* and inhibiting the expression of DELLA. ABA can influence floral primordia initiation via the interaction between *HaABF5* and *HaSOC1*. *HaMYC2s* induced downstream genes related to female flower development, such as *HaLFY*. In terms of BR, it could repress *HaFLC* expression and activated *HaSPLs*. This activation of *HaSPLs* led to the expression of *HaSOC1, HaAP1/HaFUL*, and the new *HaMIKC* to regulate the early stage of flower development. The JA signaling pathway can regulate downstream floral integrators and directly interact with other hormone signaling pathways through *HaMYC2*. There is a consensus that phytohormones can strongly affect the flowering process through the circadian clock. We confirmed that GA, IAA, and BR were crucial phytohormones required for the blooming process. Previous studies have shown that many phytohormones contribute to the expansion of decorative flowers and flower senescence by regulating the circadian clock [57,78,79,80]. Further investigation of the genes involved in these processes is necessary to gain novel insights into how the molecular clock can regulate the speed of flower development. CTK, ETH, and SA were identified as key regulators affecting flower longevity. Among them, CTK was demonstrated to significantly inhibit ETH production and delay flower senescence. More research is needed to prove potential links between JA, SA, and ETH in *H. arborescens.* Additionally, it is worth exploring the interaction between the reduced number of downstream *ERFs* and the prolonged reservation of decorative flowers during the process of flower senescence.

## 4. Materials and Methods

### 4.1. Plant Material and Phenological Observation

Perennial woody plants of *H. arborescens* ‘Annabelle’ were planted at the experimental nursery of the Beijing Forestry University (Beijing, China) at 39°56′ N latitude, 116°20′ E longitude. Plants with healthy growth and no peats or diseases were selected as the test material. The phenological observation started in March and extended through July. The flower development process was divided into several stages based on the developmental order of the shoot apical meristem and the morphological change in decorative flowers (Figure 1). To further observe the developmental characteristics, the developed apical shoots up to the visible flower bud stage were microscopically examined under a stereomicroscope (LEICA M165).

To investigate transcriptomic changes during flower development, we collected seven stages with distinct morphological changes as samples representing the process of current-year flower development. The morphological details of different stages are listed in Table 1. Samples of VS, FI, and FM were collected from vegetative buds and flower buds at the shoot apex. Samples of BS-1, BS-2, BS-3, and FS were collected from sepals of decorative florets. The materials at each stage were divided into two parts: one for a phytohormone assay, and the other for transcriptome sequencing. These samples were frozen in liquid nitrogen and stored at −80 °C until use.

### 4.2. Quantification of Phytohormones during Flower Development

The indole-3-acetic acid (IAA), gibberellins (GA_3_), zeatin riboside (ZR), abscisic acid (ABA), brassinosteroid (BR), and jasmonic acid (JA) were measured at seven flower developmental stages of *H. arborescens* ‘Annabelle’ using an indirect ELISA technique [81,82]. Approximately 0.5 g (fresh weight) of each stage was subjected to analysis. Three biological replicates were performed. After being homogenized in liquid nitrogen, samples from the seven stages were extracted in cold 80% (*v*/*v*) methanol with butylated hydroxytoluene (1 mmol/L) for 5 h at 4 °C. The extracts were collected after centrifugation at 3500 r/min (4 °C) for 8 min. The supernatant was passed through Chromosep C18 columns (C18 Sep-Park Cartridge, Waters Corp., Milford, MA, USA). The hormone fractions were prewashed with 1 mL of 80% (*v*/*v*) methanol and eluted with 5 mL of 100% (*v*/*v*) methanol, 5 mL of ether, and 5 mL of 100% (*v*/*v*) methanol from the columns. Then, they were dried under N2 and dissolved in 2 mL of phosphate-buffered saline (PBS) containing 0.1% (*v*/*v*) Tween 20 and 0.1% (*w*/*v*) gelatin for analysis via an ELISA. The samples were diluted an appropriate amount of the standard sample with PBS to 8 concentrations (including 0 ng/mL). These series of standard samples and test samples were added to the 96-well ELISA plate; then, antibodies were added and incubated at 37 °C for 30 min. After the samples had been washed four times with a PBS + Tween 20 (0.1% (*v*/*v*)) buffer, 10 mL of the diluted enzyme-linked secondary antibody was added, and the samples were incubated at 37 °C for 30 min and then washed as above. Finally, the buffered enzyme substrate (orthophenylene diamino) was added, and the enzyme reaction was carried out in the dark at 37 °C for 15 min and then terminated using 50 μL of 2 mol/L H_2_SO_4_. Finally, calculations of the enzyme immunoassay data were made as described by Weiler et al. [83]. The results were analyzed using a one-way analysis of variance (ANOVA) and the differences were compared using the IBM SPSS Statistics 27.0.1 software and Duncan’s multiple range test (*p* < 0.05). The various hormone contents of the different developmental stages are presented as the means ± SE of at three replicates.

### 4.3. RNA Extraction and RNA-seq Library Construction

According to the manufacturer’s instructions, total RNA was derived from the samples during the 7 stages of current-year flower development in *H. arborescens* ‘Annabelle’ using a Trizol reagent kit (Invitrogen, Carlsbad, CA, USA). The quality and purity of RNA were assessed on an Agilent 2100 Bioanalyzer (Agilent Technologies, Palo Alto, CA, USA) and an ND-2000 (NanoDrop Technologies) and checked using 1% RNase free agarose gel electrophoresis. Only RNA samples that passed the quality tests were used for the RNA-seq analysis.

After the total RNA was extracted, eukaryotic mRNA was enriched with Oligo(dT) beads. Then, the enriched mRNA was fragmented into short fragments using a fragmentation buffer and reversely transcribed into cDNA by using an NEB #7530 RNA Library Prep Kit for Illumina (#E7530, New England Biolabs). Then, the total mixed RNA from *H. arborescens* in the flower development process was used for cDNA library construction. The library quality was subsequently detected using the High Sensitivity DNA assay Kit (Agilent Technologies). The resulting cDNA library was sequenced using Illumina Novaseq6000 by Gene Denovo Biotechnology Co. (Guangzhou, China). To obtain high-quality data, it was further filtered using fastp (version 0.18.0). The parameters were as follows: first removing reads containing adapters, and then removing reads containing more than 10% of unknown nucleotides (N), before finally removing low-quality reads containing more than 50% low-quality (Q-value ≤ 20) bases. Then, the clean reads were assembled using Trinity to construct unique consensus sequences for a subsequent information analysis [33].

### 4.4. Functional Annotation of Unigenes and Screening for Differentially Expressed Genes

First, all unigene sequences were aligned against protein databases using BLASTX (e-value < 0.00001) in the following order: Nr protein database (https://ftp.ncbi.nlm.nih.gov/blast/db/FASTA/, accessed on 14 May 2020), SwissProt protein database (https://www.uniprot.org/, accessed on 14 May 2020), KEGG pathway database (http://www.kegg.jp/, version Release 93.0), and KOG database (https://www.ncbi.nlm.nih.gov/KOG/, accessed on 24 July 2015). We then obtained the highest similarity with a given unigene of the protein containing the function annotation information. The gene expression levels were calculated and normalized based on the fragments per kilobase per million from the mapped fragments (FPKM). A gene was defined as expressed if it had an FPKM ≥ 2 in at least one of the seven transcriptomes [84].

A transcriptome comparison analysis was performed using the DESeq2 software (version 3.12.1) between two adjacent stages (and with edgeR between two samples) [85]. Genes with the parameters of a false discovery rate (FDR) ≤ 0.05 and a |log_2_FoldChange| ≥ 1 would be considered as significant DEGs (differentially expressed genes) in the relative expression levels. To understand the high-level functions and utilities of the biological system, all DEGs were assigned to the diverse terms and pathways of the GO database and the KEGG database. We considered GO terms and KEGG pathways showing *p*-values < 0.05, *p*-values < 0.01, and *p*-values < 0.001 as significant, highly significant, and very highly significant, respectively. Following previous research, significant GO and KEGG functional pathway enrichment analyses in each comparison were performed to screen flower development-related DEGs, including the genes involved in six flowering-related pathways, as well as genes associated with the biosynthesis and signal transduction of eight phytohormones (major associated pathways are highlighted in Table S4). All selected DEGs were used to construct the gene co-expression network (GCN).

### 4.5. Construction of the Genes Co-Expression Network (GCN) and Cluster Recognition

The highest reciprocal rank (HRR)-based gene co-expression network (GCN) methodology was utilized to investigate the interactions between DEGs involved in the current-year flower development. First, based on the FPKM of the DEGs, we selected the interactions among DEGs with Pearson’s correlation coefficient (r) as a metric, in which values greater than 0.8 or less than −0.8 were considered highly correlated and were chosen for further analysis. Next, using *R version 3.3.1* (https://www.rproject.org/, accessed on 1 December 2022), we transformed raw *r* values for each relationship between DEGs into HRR, which serve as an index for the construction of the GCN [86]. Networks were generated using different HRR cut-offs, where weights of 1/5, 1/15, and 1/25 were given cut-off scores of 10, 20, and 30, respectively. Then, the visualization of the GCN was constructed using *Cytoscape 3.9.1*. A basic parameter analysis was performed using the *Cytoscape Plug-in Network Analyzer*. We used the *Cytoscape Plugin Cluster Maker* to define MCL (Markov Cluster Algorithm) functional clusters. Meanwhile, we identified that using an inflation score (*I*) parameter of 3.0 could produce the best clustering solution. In a GCN, genes are usually represented as “nodes”, whilst the lines linking individual nodes, or “edges”, represent pairwise relationships between nodes. A collection of densely connected nodes represents a “cluster”, and the entire collection of nodes, edges, and clusters forms the co-expression “network”. Often, co-expressed genes within a cluster are expected to be functionally related to genes with a similar expression pattern.

### 4.6. Module Identification, Functional Enrichment, Key Genes and transcription factors Analysis

A GO enrichment analysis was applied to assess the biological function of each cluster. Enrichment terms were deemed significant if the corrected *p*-value (FDR) was < 0.05 (expressed as sensitivity) and if there were at least 2 genes associated with the same annotation. Meaningless clusters were excluded from the GCN with fewer than 3 genes and without any significant enriched term [87,88].

To enhance the accuracy of the analysis, clusters with a similar expression pattern were grouped into one module. Most genes in each module exhibited a significant change in gene expression during the associated stage, suggesting a reliable correlation between modules and stages [89]. The top 20 ranked GO biological process terms were selected according to their *p*-value. We defined the function of each module using a combination of expression data, significantly enriched GO terms, and literature searches.

A visualization of the node and edge attributes of each module was performed in the form of a ring network using *Cytoscape 3.9.1*. The top 10 genes (including transcription factors) with the highest intra-modular connectivity were each considered as a potential “highly connected gene” (hub gene) within each module [90]. The hub genes served as representatives of each module and hold important biological significance in system analysis. Based on the identification of hub genes, we further obtained the key gene with the highest connectivity in each module. Key genes were closely associated with specific flower development stages and represented regulating centers of the specific flower development stages.

Based on the data of the GCN and the identified key genes, we further found important TFs involved in the regulation of flower development that had an absolute Pearson’s correlation coefficient value greater than or equal to 0.8 between key genes and TFs in the GCN. The co-expression networks between key genes and TFs were visualized in *Cytoscape 3.9.1*.

### 4.7. Validation of RNA-seq Data via Quantitative Real-Time PCR (qRT-PCR)

To confirm the accuracy of the transcriptome data, we selected twelve DEGs that might be related to flower development for qRT-PCR analysis. The total RNA was reverse transcribed into cDNA using the HiScript III RT SuperMix for qPCR (+gDNA wiper) from Vazyme Biotech, Nanjing, China. Three independent biological replicates were conducted for each tissue sample via real-time PCR analysis. Gene-specific primers for each candidate gene and the internal reference gene (*EF1-β*) were created using the Primer Premier 5 software (Premier Biosoft, San Francisco, CA, USA) and produced by Bioengineering Co., Ltd. (Shanghai, China) in accordance with the design principles of qRT-PCR primers (Table S9). The reaction system was prepared referring to the manufacturers’ instructions of the TB Green^®^ Premix Ex TaqTM II (Takara, Shiga, Japan), and PCR amplification was carried out. The reaction system and reaction processes are shown in Table S10. The expression levels of the DEGs were normalized and mapped using the 2^−∆∆CT^ method [91].

## Figures and Tables

**Table 1:** Samples of current -year flower development process in *H. arborescens* ‘Annabelle’.

Stage	Description	Figure
Vegetative Stage (VS)	Vegetative shoot apical meristem.	Figure 1a
Floral Initiation (FI)	Flower bud expansion: many flower primordia clearly distinguishable and forming a spherical shape.	Figure 1d
Floral Morphogenesis (FM)	Secondary branching elongation of spherical inflorescences and floral organ differentiation.	Figure 1g
Blooming Stage 1 (BS-1)	Early blooming stage: fifth branching of inflorescences formed; decorative floret displayed tender green.	Figure 1j
Blooming Stage 2 (BS-2)	Middle blooming stage: sepals of decorative florets were not fully open; the color of sepals gradually changed from green to white.	Figure 1k
Blooming Stage 3 (BS-3)	Full blooming stage: more than 80% of decorative florets were snowy-colored in inflorescence.	Figure 1l
Flower Senescence (FS)	The senescent inflorescence displayed a deep green.	Figure 1m

**Table 2:** Simple parameters of constructed gene co-expression network (GCN).

Simple Parameters			
Number of nodes	428	Clustering coefficient	0.739
Number of edges	4838	Network density	0.536
Avg. number of neighbors	26.784	Network heterogeneity	0.318
Network of diameter	3	Network centralization	0.358
Characteristic path length	1.468	Connected components	14

## Data Availability

All data generated or analyzed during this study are available.

## Supplementary Materials

The following supporting information can be downloaded at https://www.mdpi.com/article/10.3390/plants12244103/s1.
