# Supplementary material for: Hydrangea arborescens ‘Annabelle’ Flower Formation and Flowering in the Current Year
Source: Plants (Basel). 2023 Dec 7;12(24):4103. doi: 10.3390/plants12244103 (PMC10748224; doi:10.3390/plants12244103)
Supplement: Supplementary file 1 [file plants-12-04103-s001.zip › Supplementary Figures.pdf]

Supplementary Figures

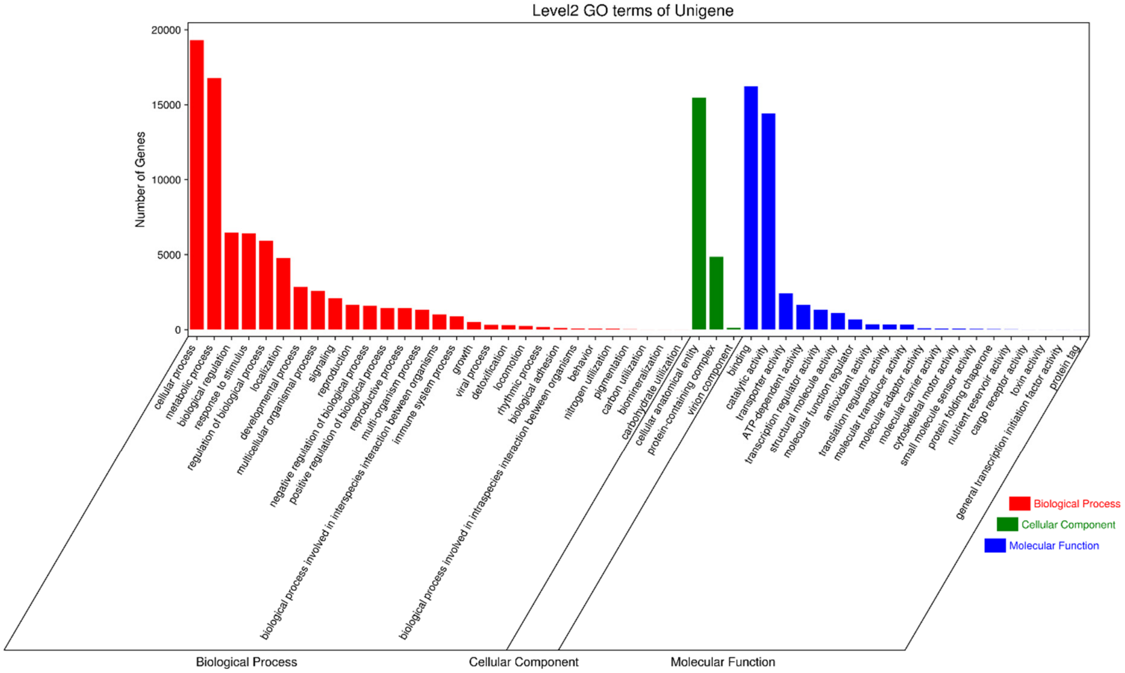

Figure S1. Gene ontology (GO) classification analysis different expression genes (DEGs) during the current-year flower development in *Hydrangea arborescens* 'Annabelle'

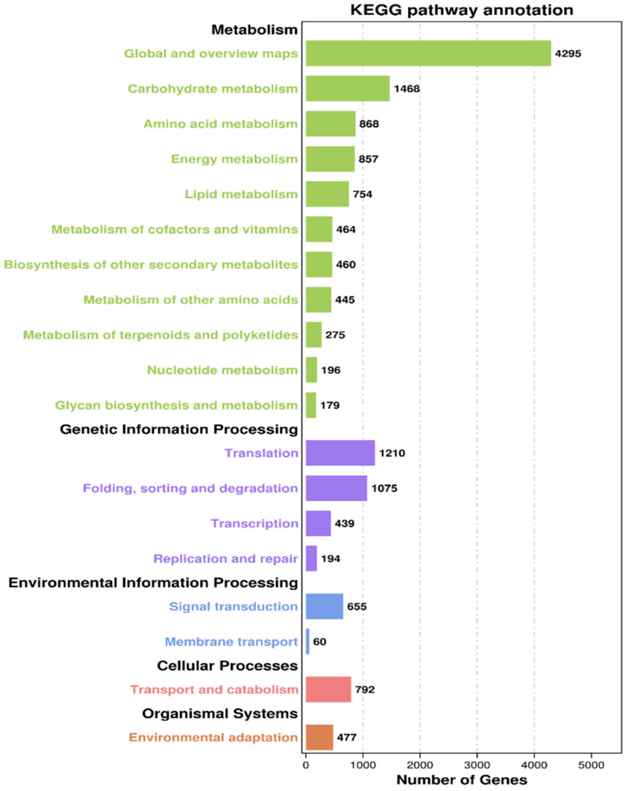

Figure S2. KEGG classification analysis different expression genes (DEGs) during the current-year flower development in *Hydrangea arborescens* 'Annabelle'

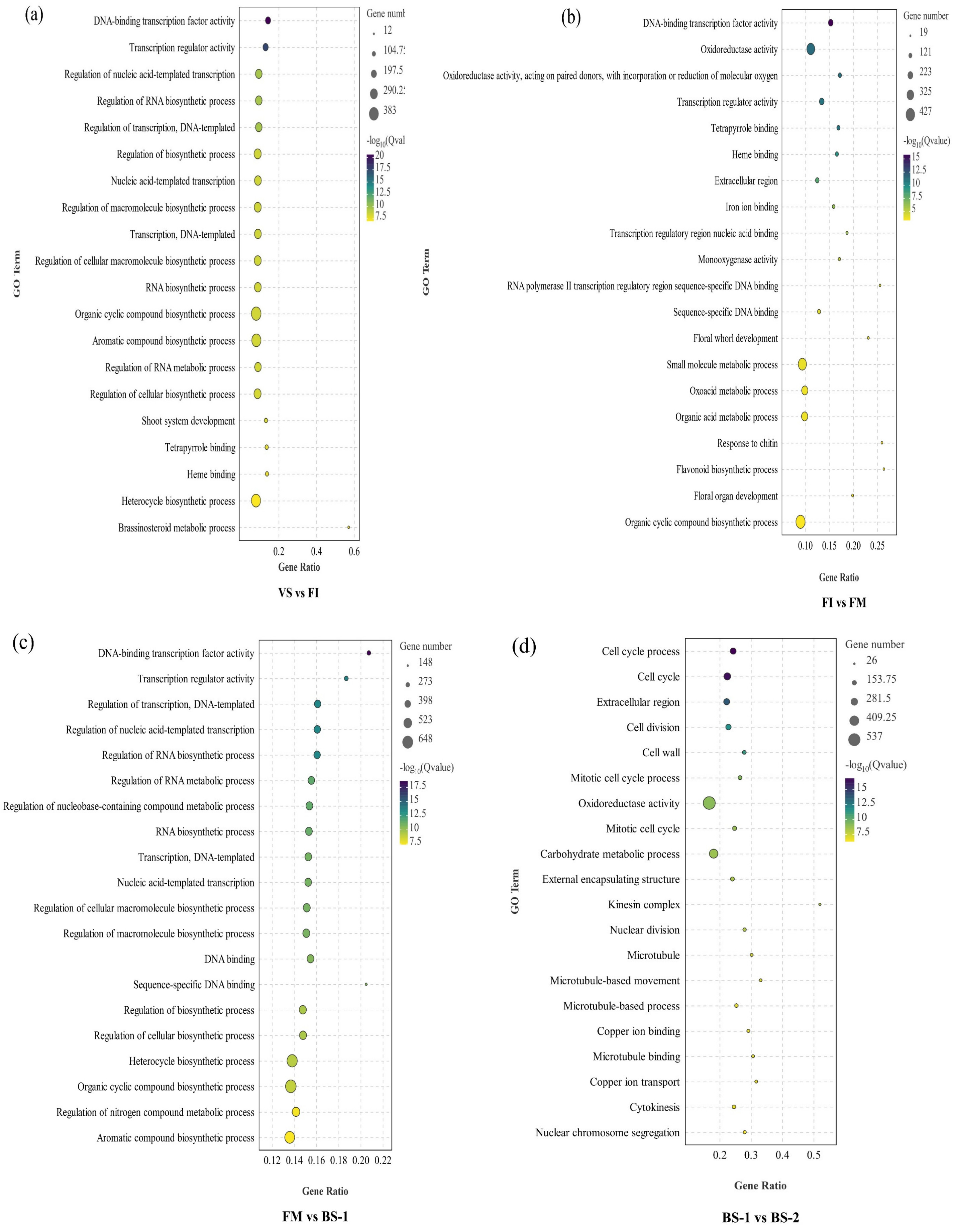

(a)
DNA-binding transcription factor activity
Transcription regulator activity
Regulation of nucleic acid-templated transcription
Regulation of RNA biosynthetic process
Regulation of transcription, DNA-templated
Regulation of biosynthetic process
Nucleic acid-templated transcription
Regulation of macromolecule biosynthetic process
Transcription, DNA-templated
Regulation of cellular macromolecule biosynthetic process
RNA biosynthetic process
Organic cyclic compound biosynthetic process
Aromatic compound biosynthetic process
Regulation of RNA metabolic process
Regulation of cellular biosynthetic process
Shoot system development
Tetrapyrrole binding
Heme binding
Heterocycle biosynthetic process
Brassinosteroid metabolic process
GO Term
0.2
0.4
0.6
Gene Ratio
Gene numb
12
197.5
383
20
17.5
15
12.5
10
7.5
VS vs FI
(b)
DNA-binding transcription factor activity
Oxidoreductase activity
Oxidoreductase activity, acting on paired donors, with incorporation or reduction of molecular oxygen
Transcription regulator activity
Tetrapyrrole binding
Heme binding
Extracellular region
Iron ion binding
Transcription regulatory region nucleic acid binding
Monooxygenase activity
RNA polymerase II transcription regulatory region sequence-specific DNA binding
Sequence-specific DNA binding
Floral whorl development
Small molecule metabolic process
Oxoacid metabolic process
Organic acid metabolic process
Response to chitin
Flavonoid biosynthetic process
Floral organ development
Organic cyclic compound biosynthetic process
GO Term
0.10
0.15
0.20
0.25
Gene Ratio
Gene number
19
121
223
325
427
$-\log_{10}$(Qvalue)
15
12.5
10
7.5
5
FI vs FM
(c)
DNA-binding transcription factor activity
Transcription regulator activity
Regulation of transcription, DNA-templated
Regulation of nucleic acid-templated transcription
Regulation of RNA biosynthetic process
Regulation of RNA metabolic process
Regulation of nucleobase-containing compound metabolic process
RNA biosynthetic process
Transcription, DNA-templated
Nucleic acid-templated transcription
Regulation of cellular macromolecule biosynthetic process
Regulation of macromolecule biosynthetic process
DNA binding
Sequence-specific DNA binding
Regulation of biosynthetic process
Regulation of cellular biosynthetic process
Heterocycle biosynthetic process
Organic cyclic compound biosynthetic process
Regulation of nitrogen compound metabolic process
Aromatic compound biosynthetic process
GO Term
0.12
0.14
0.16
0.18
0.20
0.22
Gene Ratio
Gene number
148
273
398
523
648
$-\log_{10}$(Qvalue)
17.5
15
12.5
10
7.5
FM vs BS-1
(d)
Cell cycle process
Cell cycle
Extracellular region
Cell division
Cell wall
Mitotic cell cycle process
Oxidoreductase activity
Mitotic cell cycle
Carbohydrate metabolic process
External encapsulating structure
Kinesin complex
Nuclear division
Microtubule
Microtubule-based movement
Microtubule-based process
Copper ion binding
Microtubule binding
Copper ion transport
Cytokinesis
Nuclear chromosome segregation
GO Term
0.2
0.3
0.4
0.5
Gene Ratio
Gene number
26
153.75
281.5
409.25
537
$-\log_{10}$(Qvalue)
15
12.5
10
7.5
BS-1 vs BS-2

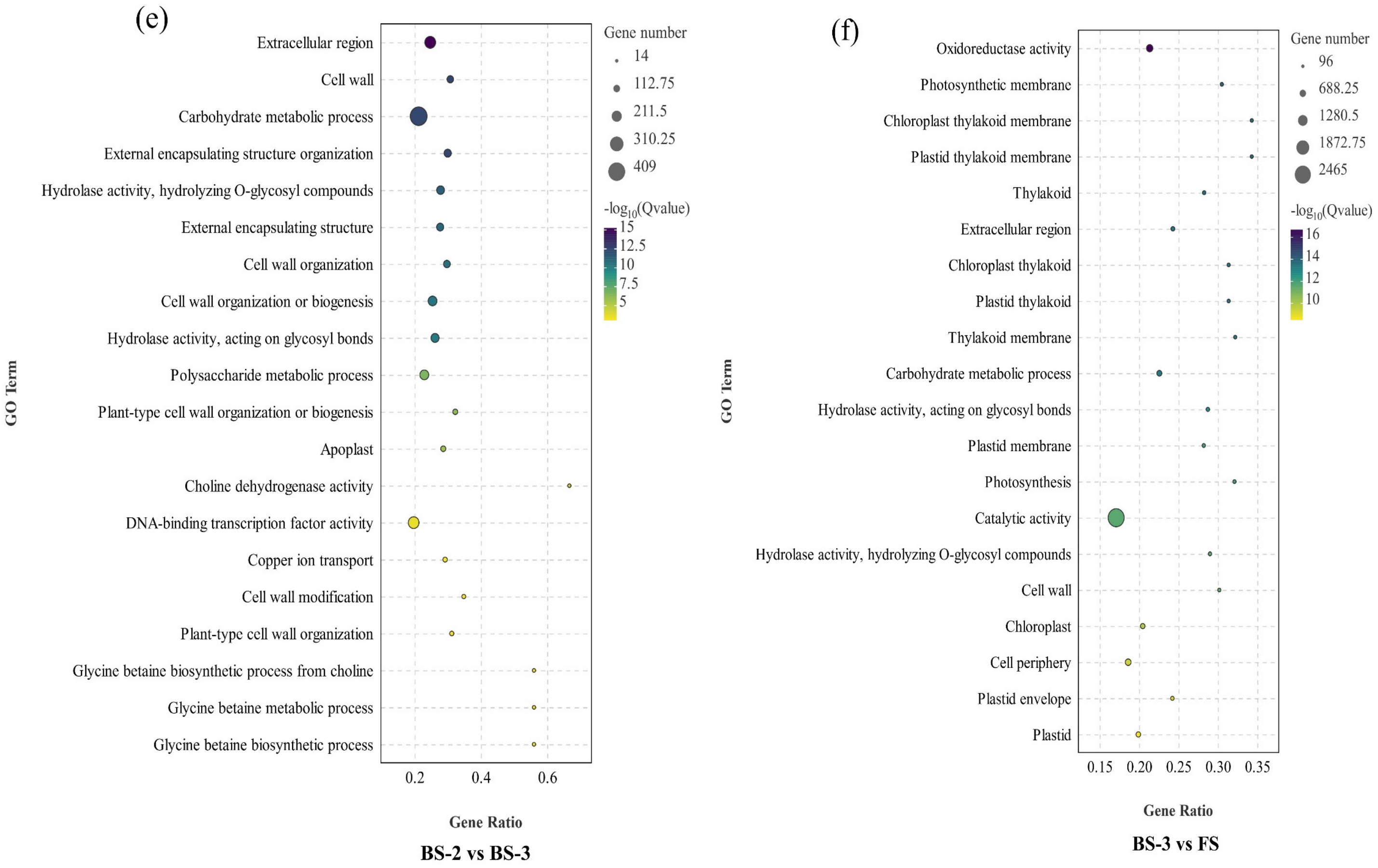

Figure S3. GO term enrichment analysis of DEGs during flower development

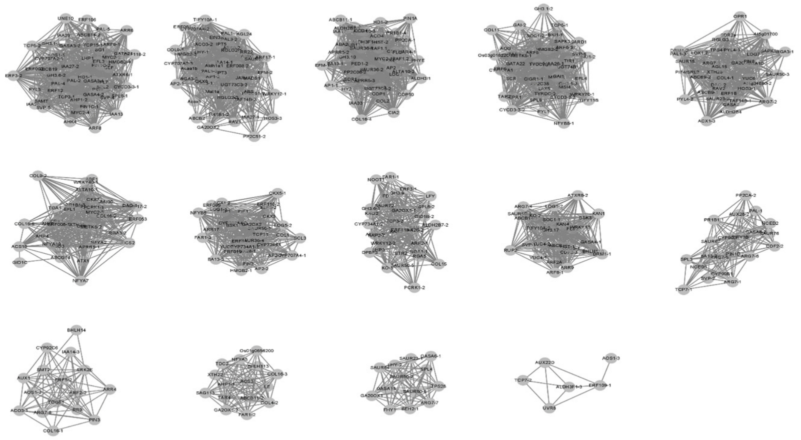

Figure S4. Summary of MCL gene co-expression network (GCN) . The network consisted of 14 biological meaningful clusters.
