# Supplementary material for: Hydrangea arborescens ‘Annabelle’ Flower Formation and Flowering in the Current Year
Source: Plants (Basel). 2023 Dec 7;12(24):4103. doi: 10.3390/plants12244103 (PMC10748224; doi:10.3390/plants12244103)
Supplement: Supplementary file 1 [file plants-12-04103-s001.zip › Supplementary Tables.pdf]

Supplementary Tables

Table S1. Statistics of the quality of the sequencing data

Sample	Raw Reads	Raw Bases	Clean Reads	Clean Bases	Q30 (%)	GC (%)
VS	46.74M	7.01G	46.54M	6.96G	93.94	44.63
FI	40.91M	6.14G	40.74M	6.09G	93.89	44.37
FM	46.87M	7.03G	46.67M	6.97G	94.01	44.69
BS-1	44.06M	6.61G	43.87M	6.55G	94.23	44.68
BS-2	41.63M	6.24G	41.46M	6.18G	94.53	44.62
BS-3	51.91M	7.79G	51.72M	7.72G	93.85	44.79
FS	52.85M	7.93G	52.65M	7.86G	94.13	45.05

Table S2. Assembly statistics of the clean reads

Unigenes Num	GC percentage(%)	N50 number	N50 length	Max length	Min length	Average length
72,614	39.85	12,481	1,828	69,246	201	1,043

Table S3. Functional annotation statistics to four databases

Database	Number	Percentage (%)
Nr	35,109	48.35
KEGG	34,367	47.33
KOG	20,697	28.50
Swiss-Prot	25,616	35.28

Table S4. The top 20 pathway of KEGG enrichment analysis

Comparison between VS and FI stages

Pathway_ID	Pathway	TEST	P-value	FDR
ko00905	Brassinosteroid biosynthesis	9	3.13E-09	3.64E-07
ko01110	Biosynthesis of secondary metabolites	173	2.10E-06	1.22E-04
ko01100	Metabolic pathways	276	2.15E-05	6.02E-04
ko00073	Cutin, suberine and wax biosynthesis	11	2.30E-05	6.02E-04
ko00904	Diterpenoid biosynthesis	10	2.60E-05	6.02E-04
ko00941	Flavonoid biosynthesis	12	0.000418712	7.78E-03
ko00940	Phenylpropanoid biosynthesis	25	0.000469385	7.78E-03
ko00909	Sesquiterpenoid and triterpenoid biosynthesis	7	0.000574501	8.33E-03
ko00380	Tryptophan metabolism	16	0.001201258	1.55E-02
ko04075	Plant hormone signal transduction	37	0.002168271	2.32E-02
ko00908	Zeatin biosynthesis	6	0.002202735	2.32E-02
ko00195	Photosynthesis	14	0.003413644	3.30E-02
ko00906	Carotenoid biosynthesis	7	0.008398379	7.49E-02
ko00945	Stilbenoid, diarylheptanoid and gingerol biosynthesis	7	0.01908082	1.49E-01
ko00460	Cyanoamino acid metabolism	9	0.01926321	1.49E-01
ko00240	Pyrimidine metabolism	10	0.02236994	1.62E-01
ko00901	Indole alkaloid biosynthesis	2	0.04092927	2.73E-01
ko00330	Arginine and proline metabolism	11	0.0454186	2.73E-01
ko00430	Taurine and hypotaurine metabolism	3	0.04888333	2.73E-01
ko04141	Protein processing in endoplasmic reticulum	35	0.0491084	2.73E-01

Comparison between FI and FM stages

Pathway_ID	Pathway	TEST	P-value	FDR
ko01100	Metabolic pathways	432	3.13E-09	3.64E-07
ko01110	Biosynthesis of secondary metabolites	249	2.10E-06	1.22E-04
ko00196	Photosynthesis - antenna proteins	20	2.15E-05	6.02E-04
ko00941	Flavonoid biosynthesis	20	2.30E-05	6.02E-04
ko00630	Glyoxylate and dicarboxylate metabolism	30	2.60E-05	6.02E-04
ko00270	Cysteine and methionine metabolism	34	0.000418712	7.78E-03
ko04146	Peroxisome	31	0.000469385	7.78E-03
ko00940	Phenylpropanoid biosynthesis	32	0.000574501	8.33E-03
ko00999	Biosynthesis of various plant secondary metabolites	18	0.001201258	1.55E-02
ko00195	Photosynthesis	19	0.002168271	2.32E-02
ko00260	Glycine, serine and threonine metabolism	20	0.002202735	2.32E-02
ko00945	Stilbenoid, diarylheptanoid and gingerol biosynthesis	11	0.003413644	3.30E-02
ko00073	Cutin, suberine and wax biosynthesis	10	0.008398379	7.49E-02
ko01040	Biosynthesis of unsaturated fatty acids	14	0.01908082	1.49E-01
ko04075	Plant hormone signal transduction	48	0.01926321	1.49E-01
ko00910	Nitrogen metabolism	13	0.02236994	1.62E-01
ko01200	Carbon metabolism	70	0.04092927	2.73E-01
ko04016	MAPK signaling pathway - plant	33	0.0454186	2.73E-01

ko00053	Ascorbate and aldarate metabolism	20	0.04888333	2.73E-01
ko00906	Carotenoid biosynthesis	9	0.0491084	2.73E-01
ko00250	Alanine, aspartate, and glutamate metabolism	16	0.00487	2.78E-02
ko00360	Phenylalanine metabolism	14	0.005177	2.82E-02
ko00380	Tryptophan metabolism	18	0.00578	3.02E-02
ko00350	Tyrosine metabolism	17	0.006428	3.21E-02
ko00950	Isoquinoline alkaloid biosynthesis	9	0.010869	5.22E-02
ko00480	Glutathione metabolism	25	0.012594	5.66E-02
ko00710	Carbon fixation in photosynthetic organisms	23	0.012733	5.66E-02
ko00410	beta-Alanine metabolism	15	0.014673	6.29E-02
ko00909	Sesquiterpenoid and triterpenoid biosynthesis	6	0.015892	6.58E-02
ko00904	Diterpenoid biosynthesis	7	0.022473	8.91E-02

Comparison between FM and BS-1 stages

Pathway_ID	Pathway	TEST	P-value	FDR
ko00196	Photosynthesis - antenna proteins	24	3.72E-09	4.76E-07
ko00941	Flavonoid biosynthesis	24	3.01E-08	1.59E-06
ko00940	Phenylpropanoid biosynthesis	49	4.37E-08	1.59E-06
ko01100	Metabolic pathways	494	4.96E-08	1.59E-06
ko03030	DNA replication	21	5.00E-07	1.28E-05
ko00904	Diterpenoid biosynthesis	15	1.28E-06	2.73E-05
ko01110	Biosynthesis of secondary metabolites	287	5.57E-06	1.02E-04
ko00945	Stilbenoid, diarylheptanoid and gingerol biosynthesis	16	1.54E-05	2.47E-04
ko00073	Cutin, suberine and wax biosynthesis	14	7.24E-05	1.03E-03
ko00908	Zeatin biosynthesis	10	8.98E-05	1.15E-03
ko00040	Pentose and glucuronate interconversions	28	0.000106	1.24E-03
ko04075	Plant hormone signal transduction	64	0.000181	1.93E-03
ko00195	Photosynthesis	24	0.000233	2.30E-03
ko00380	Tryptophan metabolism	25	0.000424	3.87E-03
ko04626	Plant-pathogen interaction	62	0.000621	5.30E-03
ko04016	MAPK signaling pathway - plant	43	0.000889	7.11E-03
ko00905	Brassinosteroid biosynthesis	6	0.001006	7.57E-03
ko00906	Carotenoid biosynthesis	11	0.002552	1.81E-02
ko00410	beta-Alanine metabolism	20	0.003746	2.52E-02
ko00053	Ascorbate and aldarate metabolism	24	0.005079	3.25E-02

Comparison between BS-1 and BS-2 stages

Pathway_ID	Pathway	TEST	P-value	FDR
ko00940	Phenylpropanoid biosynthesis	70	3.56E-12	2.93E-10
ko00196	Photosynthesis - antenna proteins	32	4.81E-12	2.93E-10
ko01100	Metabolic pathways	674	5.25E-09	2.13E-07
ko00195	Photosynthesis	37	1.61E-07	4.91E-06
ko01110	Biosynthesis of secondary metabolites	393	3.51E-07	8.57E-06
ko03030	DNA replication	25	4.95E-07	1.01E-05

ko00710	Carbon fixation in photosynthetic organisms	48	5.99E-06	1.04E-04
ko00941	Flavonoid biosynthesis	23	4.14E-05	6.31E-04
ko00073	Cutin, suberine and wax biosynthesis	15	0.000675	9.15E-03
ko00945	Stilbenoid, diarylheptanoid and gingerol biosynthesis	15	0.002327	2.84E-02
ko00910	Nitrogen metabolism	19	0.002588	2.87E-02
ko00062	Fatty acid elongation	15	0.002898	2.95E-02
ko00901	Indole alkaloid biosynthesis	4	0.004699	4.07E-02
ko00480	Glutathione metabolism	42	0.004739	4.07E-02
ko00270	Cysteine and methionine metabolism	46	0.00501	4.07E-02
ko00360	Phenylalanine metabolism	21	0.005561	4.24E-02
ko01040	Biosynthesis of unsaturated fatty acids	19	0.006996	5.02E-02
ko00909	Sesquiterpenoid and triterpenoid biosynthesis	9	0.009356	6.34E-02
ko00950	Isoquinoline alkaloid biosynthesis	13	0.013495	8.67E-02
ko01230	Biosynthesis of amino acids	84	0.019407	1.13E-01

Comparison between BS-2 and BS-3 stages

Pathway_ID	Pathway	TEST	P-value	FDR
ko03010	Ribosome	151	7.95E-10	1.02E-07
ko00040	Pentose and glucuronate interconversions	41	1.92E-07	1.23E-05
ko00073	Cutin, suberine and wax biosynthesis	19	1.93E-06	8.23E-05
ko00940	Phenylpropanoid biosynthesis	54	7.13E-06	2.28E-04
ko01100	Metabolic pathways	632	6.51E-05	1.67E-03
ko01110	Biosynthesis of secondary metabolites	357	0.001643	3.51E-02
ko00906	Carotenoid biosynthesis	13	0.003377	6.17E-02
ko00908	Zeatin biosynthesis	9	0.004646	7.43E-02
ko00945	Stilbenoid, diarylheptanoid and gingerol biosynthesis	14	0.005368	7.63E-02
ko00910	Nitrogen metabolism	17	0.01146	1.47E-01
ko00500	Starch and sucrose metabolism	51	0.020133	2.34E-01
ko00941	Flavonoid biosynthesis	16	0.024361	2.46E-01
ko00905	Brassinosteroid biosynthesis	5	0.024945	2.46E-01
ko00999	Biosynthesis of various plant secondary metabolites	21	0.02862	2.62E-01
ko00520	Amino sugar and nucleotide sugar metabolism	41	0.033399	2.73E-01
ko00460	Cyanoamino acid metabolism	16	0.035224	2.73E-01
ko00750	Vitamin B6 metabolism	6	0.038246	2.73E-01
ko00901	Indole alkaloid biosynthesis	3	0.038388	2.73E-01
ko00430	Taurine and hypotaurine metabolism	5	0.04598	2.96E-01
ko00195	Photosynthesis	22	0.046267	2.96E-01

Comparison between BS-3 and FS stages

Pathway_ID	Pathway	TEST	P-value	FDR
ko01100	Metabolic pathways	779	1.34E-30	1.75E-28
ko01110	Biosynthesis of secondary metabolites	447	3.51E-16	2.30E-14
ko00195	Photosynthesis	49	1.88E-14	8.22E-13
ko00196	Photosynthesis - antenna proteins	32	8.70E-12	2.85E-10

ko00999	Biosynthesis of various plant secondary metabolites	35	1.48E-07	3.87E-06
ko00073	Cutin, suberine and wax biosynthesis	20	7.38E-07	1.61E-05
ko00040	Pentose and glucuronate interconversions	39	4.39E-06	7.85E-05
ko04075	Plant hormone signal transduction	91	4.79E-06	7.85E-05
ko00904	Diterpenoid biosynthesis	16	2.08E-05	3.03E-04
ko00500	Starch and sucrose metabolism	64	6.81E-05	8.92E-04
ko00460	Cyanoamino acid metabolism	23	0.000122667	1.46E-03
ko00909	Sesquiterpenoid and triterpenoid biosynthesis	12	0.000165504	1.81E-03
ko00350	Tyrosine metabolism	31	0.000220656	2.22E-03
ko00260	Glycine, serine and threonine metabolism	32	0.000363227	3.40E-03
ko00905	Brassinosteroid biosynthesis	7	0.000995308	8.69E-03
ko00630	Glyoxylate and dicarboxylate metabolism	41	0.001642304	1.34E-02
ko00910	Nitrogen metabolism	19	0.003317366	2.56E-02
ko00710	Carbon fixation in photosynthetic organisms	37	0.0150878	1.10E-01
ko00360	Phenylalanine metabolism	19	0.02859593	1.87E-01
ko00130	Ubiquinone and other terpenoid-quinone biosynthesis	17	0.02859746	1.87E-01

Table S9. The Gene-specific primer sequences used in qRT-PCR.

Gene Name	Forward Primer Sequence (5′–3′)	Reverse Primer Sequence (5′–3′)
EF1-β	CGCAGCTGTTTTAGGGAAGCC	GCGAGCTGCGAAGACACAGA
FLC	AGAGCCAAATGGTGAGCAACT	GCAGCAATCTCCTTTGCGAG
CLF	TTTGCTATCGGCTGGTGATAA	TCCAGTACCGTCCGTAGAAG
ARF2-2	ATACTTGTATGAACCCACTG	CTCCACCTGATTATTAGCAC
SPL4	GTACCACCGTAGGCACAAGG	TCTTCCTACGCCGCTCATTG
SPL6-2	GGTTGATTGGAGGGAGACCG	GCCGTTCGGGATCTCTTTGA
FD	TACTAAAGACCCTCCGGCGA	CGGGTCGGAGCTTCCAATAA
LFY	CCGGATTTCTTGGCGTACCT	TGAGCAGTGCCGTGATTTCT
SOC1-2	GCGTATAGAGAACGCGACGA	TCAGCATCGCAAAGAACCGA
COL2	TGTGCCAGTTCAGTGTGGAG	GTGACTGATTGGAGCAGGGT
AGL15	GAAGGGTTATTGTCGGTGAA	TGTCGGCGTAATGTCTCG
GAI1	ACAGAGGGTTCGTCGCTAA	GCTCCACCATCGTCAATAT

Table S10. The reaction system and reaction processes used for qRT-PCR.

The Reaction System		The Reaction Process		
TB Green Premix Ex Taq II	10μL	Pre-denaturation	95℃	1 cycle
Forward Primer	0.4μL		95℃	
Reverse Primer	0.4μL	PCR reaction	60℃	40 cycles
cDNA	2μL		72℃	
Total volume	20μL	Melting curve	60-95℃	
